# Comprehensive Analysis of the Transcriptome-Wide m6A Methylome of Heart via MeRIP After Birth: Day 0 vs. Day 7

**DOI:** 10.3389/fcvm.2021.633631

**Published:** 2021-03-22

**Authors:** Chuanxi Yang, Kun Zhao, Jing Zhang, Xiaoguang Wu, Wei Sun, Xiangqing Kong, Jing Shi

**Affiliations:** ^1^Department of Cardiology, Medical School of Southeast University, Nanjing, China; ^2^Department of Cardiology, The First Affiliated Hospital of Nanjing Medical University, Nanjing, China

**Keywords:** m6A, epitranscriptome, heart regeneration, METTL3, cardiomyocyte

## Abstract

**Aim:** To systematically classify the profile of the RNA m6A modification landscape of neonatal heart regeneration.

**Materials and Methods:** Cardiomyocyte proliferation markers were detected via immunostaining. The expression of m6A modification regulators was detected using quantitative real-time PCR (qPCR) and Western blotting. Genome-wide profiling of methylation-modified transcripts was conducted with methylation-modified RNA immunoprecipitation sequencing (m6A-RIP-seq) and RNA sequencing (RNA-seq). The Gene Expression Omnibus database (GEO) dataset was used to verify the hub genes.

**Results:** METTL3 and the level of m6A modification in total RNA was lower in P7 rat hearts than in P0 ones. In all, 1,637 methylation peaks were differentially expressed using m6A-RIP-seq, with 84 upregulated and 1,553 downregulated. Furthermore, conjoint analyses of m6A-RIP-seq, RNA-seq, and GEO data generated eight potential hub genes with differentially expressed hypermethylated or hypomethylated m6A levels.

**Conclusion:** Our data provided novel information on m6A modification changes between Day 0 and Day 7 cardiomyocytes, which identified that increased METTL3 expression may enhance the proliferative capacity of neonatal cardiomyocytes, providing a theoretical basis for future clinical studies on the direct regulation of m6A in the proliferative capacity of cardiomyocytes.

## Introduction

Myocardial infarction, a leading cause of death worldwide, is characterized by a significant loss of cardiomyocytes and massive replacement of fibrotic tissue (1, 2). Adult mammalian cardiomyocytes have long been thought to lose their mitotic ability shortly after birth, resulting in cell cycle stagnation (3). Bergmann et al. demonstrated that adult cardiomyocytes still show limited regeneration, ranging from 0.3 to 1% per year. However, for adult mammals, including humans, myocardial injury-induced replacement of cardiomyocytes is not sufficient to restore the contractile function of the injured heart. In addition, the mammalian mouse heart exhibits excellent regenerative ability in the early neonatal stages (P0-P3), but this is no longer seen after P7 (4). This difference has prompted interest in finding the key factor relevant to cardiomyocyte proliferation.

A range of studies have attempted to regulate the cyclins, exocrine factors, and transcription factors involved in cardiomyocyte proliferation to promote cardiac regeneration under pathological or physiological conditions (5–7). Although only limited effects have been found, these results strongly suggest that regulation of the cell cycle associated with myocardial proliferation may be a therapeutic strategy for cardiac repair. In particular, during cardiac development, dynamic epigenetic regulation can directly control the ability of cardiomyocytes to proliferate (8, 9). Due to the complexity and diversity of the regulation of the myocardial cell cycle, therapeutic means of extensive control of the myocardial cell cycle are still lacking.

The biological value of reversible RNA methylation has only recently been discovered, well after that of DNA methylation and histone modification (10, 11). m6A (N6-methyladenosine) modification, an important part of RNA epigenetics, is the most common and abundant internal modification in eukaryotic mRNA, miRNA, long non-coding RNA (lncRNA), and circular RNA (12, 13). Its continuous and dynamic regulation may have a profound impact on various biological processes in mammals, including the maintenance and differentiation of embryonic stem cells and the regulation of the cell cycle through governing mRNA stability, maturation, splicing, transport, and translation (14–16).

Although recent studies have shown that the epigenetic regulation of m6A is closely related to cardiovascular diseases, including cardiac arrhythmia (17), coronary heart disease (18), and cardiomyocyte hypertrophy (19), whether it is also involved in the process of cardiac regeneration remains unknown. In this study, we tested the level of m6A in the neonatal stages P1 and P7 and explored a new layer of epigenetic change in the genome-wide screening of altered methylation-modified transcript profiles in cardiomyocyte regeneration. Thus, we sought to further investigate whether it is a possible therapeutic target for the promotion of cardiac regeneration.

## Materials and Methods

### Sample Collection

Heart tissue from P0 and P7 Sprague-Dawley rats after birth were obtained from Charles River Laboratories. After the rats were sacrificed via 2% isoflurane anesthesia, their hearts were quickly removed from the chest, washed three times in phosphate buffer saline at 4°C, and separated into centrifuge tubes. All tissues were stored at −80°C before use. All procedures were approved by the Experimental Animal Care and Use Committee of Nanjing Medical University and conducted in accordance with the guide for the Care and Use of Laboratory Animals (NIH publication no. 85–23, revised 1996), under the approval number IACUC-1712010.

### Cell Culture

Sprague-Dawley rats, both 0 days old and 7 days old (Charles River Laboratories) were obtained from the Experimental Animal Center of Nanjing Medical University. Neonatal rat cardiac myocytes (NRCMs) were prepared as previously described. In brief, the rats were anesthetized with 2% isoflurane to induce sacrifice, and the ventricular tissue was obtained soon thereafter. Subsequently, the cardiac myocytes were dispersed by incubation at 37°C with collagenase II (Worthington, Lakewood, NJ) and pancreatin (Sigma, St. Louis, MO). The cell suspension was centrifuged through a discontinuous Percoll gradient (Amersham Pharmacia Biotech, Uppsala, Sweden), and the cardiomyocytes were carefully removed from the gradient. The cardiomyocytes were initially grown in plating cardiac myocyte medium (ScienCell, Carlsbad, USA). The cardiomyocytes were harvested for further study 2 days after culturing.

Transfection of Methyltransferase-like 3 (METTL3) siRNA (5′-GCACUUGGACUUAAGGAAUTT-3′) /scrambled controls (RiboBio, Guangzhou, China) was initiated using Lipofectamine RNAiMAX (Invitrogen, Carlsbad, CA, USA). Transfection procedures were carried out following the manufacturer's instructions when the cells were 60–70% confluent. In addition, recombinant adenovirus expressing rat METTL3 (2 × 10^7^ pfu/mL) (GeneChem) was used to infected cells. After 48 h, the cells received other treatment or were scratched.

### Western Blotting Analyses

NRCMs and heart tissues were lysed in RIPA buffer (P0013C, Beyotime), supplemented with 1 mM PMSF (ST505, Beyotime). After denaturing, 30 mg total protein was subjected to electrophoresis in 10% sodium dodecyl sulfate-polyacrylamide gel electrophoresis (SDS-PAGE) gels and transferred to polyvinylidene fluoride (PVDF) membranes (Merck-Millipore, Shanghai, China), and 5% bovine serum albumin in TBS-Tween was used to block for 1 h. Then the proteins were incubated with the following primary antibodies at 4°C overnight: anti-Proliferating cell nuclear antigen (PCNA), anti-Methyltransferase-like 3 (METTL3), anti-Methyltransferase-like 14 (METTL14), anti-Wilms tumor 1 associated protein (WTAP), anti-Fat-mass and obesity-related proteins (FTO), anti-ALKB homolog 5 (ALKBH5), anti-Ankyrin-2 (Ank2), anti-Cardiomyopathy associated 5 (Cmya5), anti-F-box protein 32 (Fbxo32), anti- 6-phosphofructo-2-kinase/fructose-2,6-biphosphatase 2 (Pfkfb2), anti-24-dehydrocholesterol reductase (Dhcr24), anti-NAC alpha domain containing (Nacad), anti-Solute carrier family 16 member 3 (Slc16a3), and anti-Solute carrier family 7, member 5 (Slc7a5). GAPDH was used as a loading control, followed by washing three times with TBS-Tween. The proteins were incubated with the corresponding secondary antibody conjugated to horseradish peroxidase and then subjected to enhanced chemiluminescence to detect the protein bands.

### RNA Extraction and Quantitative Real-Time PCR

Total RNA (exclude rRNA and tRNAs) isolation from tissue and NRCMs was performed using the TRIzol reagent (Invitrogen Inc., San Diego, CA, USA) and a RNeasy Plus Universal Mini Kit (73404, QIAGEN), following the manufacturer's instructions. RNA was reversed using random hexamer priming (Applied Biosystems). q-PCR was performed using SYBR Green reagent (Applied Biosystems) and the primers shown in Supplementary Table 1 on the ABI Prism 7500 Sequence Detection system. GAPDH was used as an internal control. The relative levels of gene expression were calculated using the 2^−ΔΔCt^ method.

### Immunostaining

The frozen sections of the heart (4 μm) from the P0 and P7 rats or the cells were fixed in 4% paraformaldehyde solution at room temperature for 12 min and washed three times with PBS. Then 10% normal goat serum and 5% bovine serum albumin in 1X PBS was used to block the sections or the cells for 1 h at room temperature. The sections were incubated overnight at 4°C with primary antibodies as follows: anti-Ki67 (Abcam, ab16667, 1:200), anti-α-Actinin (Cell Signaling Technology, Danvers, MA, USA, 69758, 1:200), anti-Aurora B (Abcam, ab2254, 1:200), and anti-pH3 (Cell Signaling Technology, Danvers, MA, USA, 69758, 1:500). After washing three times with PBS, the sections were incubated with Alexa-488- or Alexa-cy3-conjugated secondary antibodies at room temperature for 1 h followed by washing three times with PBS. Finally, microscopic images were obtained using a fluorescent microscope (Carl Zeiss, Oberkochen, Germany). The positive percentages of Ki67, Aurora B, and pH3 were quantified using NIH Image J software (MD, USA).

### Quantification of m6A in Total RNA

Total RNA from the P0 and P7 heart tissues and cardiomyocytes was used to quantify m6A modifications using an m6A RNA Methylation Quantification Kit (P-9005, EpiGentek), as previously described. Briefly, a standard curve was prepared with six concentrations ranging from 0.01 to 0.5 ng/μL m6A, as recommended by the manufacturer, and a negative sample was prepared as the control. First, 2 μL positive control, 2 μL negative control, or 300 ng total RNA was added to 96-well plates for binding. Second, the binding solution was removed from each well, after which the wells were rinsed three times with washing buffer. Third, the capture anti-m6A antibody was diluted and added to each well, and washed four times. Fourth, after the developer solution and stop solution was added, the sample was analyzed using a Nano 2000 microplate reader at 450 nm. The absolute amount of m6A was quantified, and the percentage of m6A in total RNA was established.

### MeRIP Sequencing, RNA Sequencing, and Bioinformatics Analyses

MeRIP sequencing and RNA sequencing were conducted by Guangzhou Epibiotek Co., Ltd. In brief, total RNA from three pairs of heart tissues (50–100 mg) was isolated using TRIzol reagent (Invitrogen) according to the manufacturer's protocol. Then 2 μl 10X RNA Fragmentation Buffer (100 mM Tris-HCl, 100 mM ZnCl2 in nuclease-free H_2_O) was added to 18 μg total RNA and incubated for ~5–6 min at 70°C (20). The reaction was stopped by adding 2 μl 0.5 M EDTA. A portion of the purified RNA was kept as input. The remainder of the purified RNA was incubated with 2 μg anti-m6A antibody (Abcam, cat. no. ab151230) in an IP reaction system containing Dynabeads Protein A (Invitrogen^TM^, cat. no. 10002D) and Dynabeads Protein G (Invitrogen^TM^, cat. no. 10004D) at 4°C overnight with gentle shaking. The supernatant was removed by magnetic separation and dissolved with 5 × precipitation buffer and RNA enzyme inhibitor at 4°C for 1–3 h. A low-salt precipitation buffer was used for washing two or three times, followed by another two to three washes using a high-salt precipitation buffer. Then the m6A-antibody immunoprecipitated RNA fragments were separated from the Dynabeads in a 200 μL elution buffer at 50°C for 30 min. The mRNA was extracted by phenol-chloroform and was precipitated by ethanol. Ribosomal RNA was removed from the products, and the first strand of cDNA was synthesized by SMART. PCR amplification and library purification were used to obtain an ultramicro-RNA methylated m6A library. Bioptic QseP70 Analyzer was used to conduct quality control over the library. Finally, NovaSeq's high-throughput sequencing platform and PE150 sequencing mode were used for sequencing.

To further explore the essential role of m6A mRNA modification in heart regeneration, the identified m6A peaks and Differentially expressed genes (DEGs) were subjected to network analyses (https://portal.genego.com) as previously described (21).

### Public Databases and Analyses

RNA sequencing data (GSE154071, GSE121308, GSE69855, GSE119530, and GSE123863) were downloaded from NCBI Gene Expression Omnibus (GEO, https://www.ncbi.nlm.nih.gov/gds/). Selected genes from our study were searched within these databases. Then the expression levels of the genes were analyzed using R software (version 4.0.2, www.r-project.org). The three databases were used to confirm the significant genes for MeRIP sequencing and RNA sequencing.

### EdU Staining Assessment

EdU staining was conducted using the BeyoClick™ EdU Cell Proliferation Kit with Alexa Fluor 594 (Beyotime, China, Cat. No. C00788L) according to the manufacturer's instructions. In brief, the cells were incubated with fresh DMEM added with 10 μM EdU for 24 h at 37°C/5% CO_2_. After washed with PBS three times, the cells were fixed in 4% paraformaldehyde for 15 min and permeabilizated with 0.5% Triton for 5 min at room temperature. The cells were stained with DAPI and then observed by the Zeiss fluorescence inverted microscope (Carl Zeiss, Jena, Germany) in more than three random fields of views blindly.

### Transcript Stability Assays

To elucidate whether si-METTL3 could affect the stability of the identified target genes identified by the bioinformatics analyses, we evaluated the respective transcript stability *in vivo*. Cells were incubated with Actinomycin D (20 μg/ml; GlpBio Technology, CA, USA) for 3 and 6 h to inhibit *de novo* RNA synthesis (22). Then, the total RNAs was isolated. Relative transcript levels of the identified target genes were determined by qRT-PCR. At least three independent experiments were performed for each gene of interest.

### Statistical Analyses

Clean reads were aligned to genome reference sequences using HISAT2 software. The MeRIP-enriched regions (peaks) were annotated according to the overlapping gene using the newest UCSC RefSeq database. Significant MeRIP-enriched regions (peaks) were identified for each sample at fold change ≥2. Correlations between significantly MeRIP-enriched regions (peaks) and RNA-seq data were identified for all data at fold change ≥2, with *P* < 0.05. Differentially expressed genes (DEGs) and differential m6A peaks are marked. The m6A peak was normalized to RNA-seq data (input) by exomePeak R package. Student's *t*-test was used to compare relative gene expression between P0 and P7. *P* < 0.05 was considered to indicate statistical significance. GO and KEGG pathway analyses were performed using the significantly methylated protein-coding genes and differentially expressed genes.

## Results

### m6A Is Lower in P7 Than in P0 Rats

**Figure 1:**
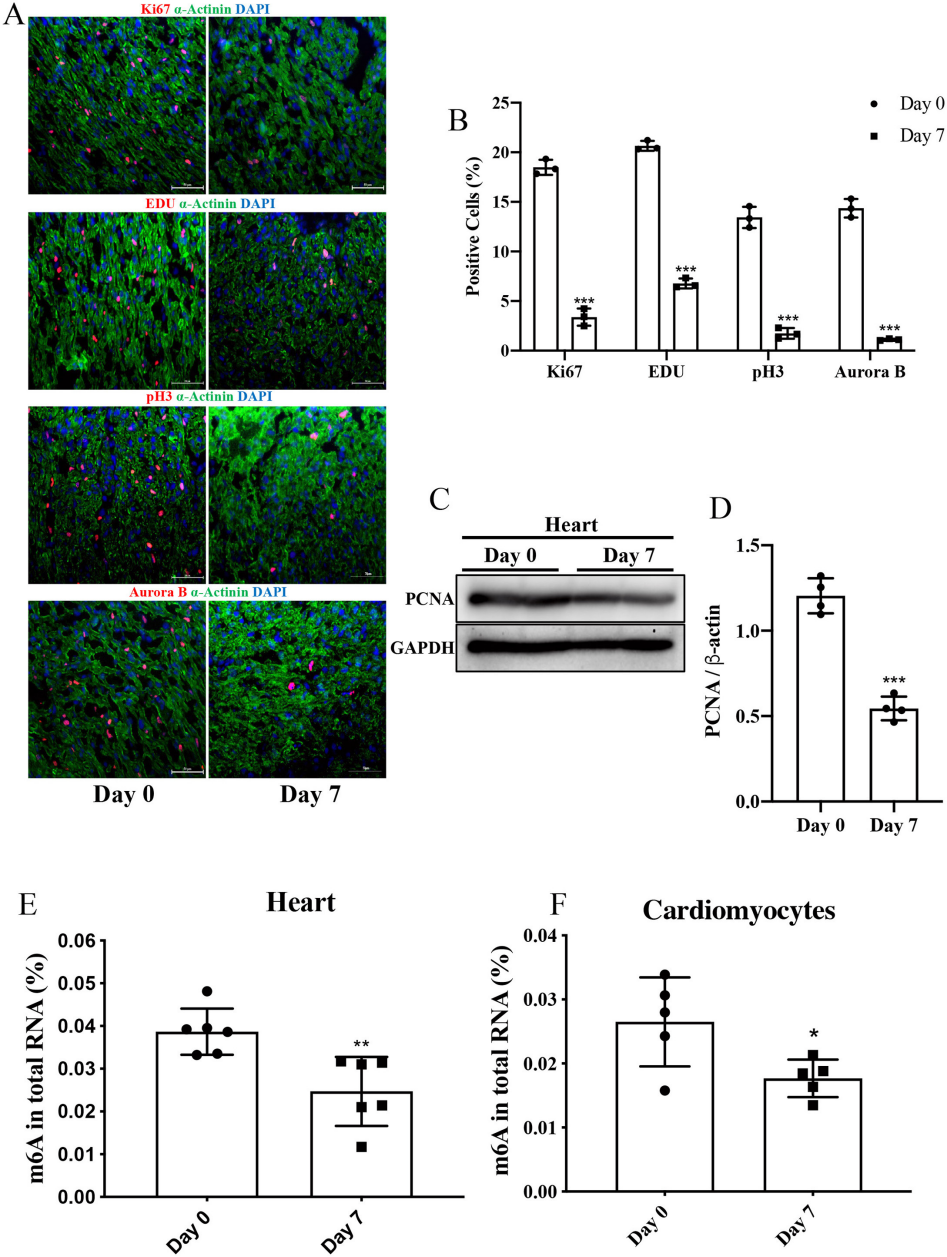
The level of m6A in P7 rat heart was decreased compared to P0. **(A,B)** Representative immunofluorescence images of paraffin-embedded heart sections labeled with α-Actinin, Ki67, pH3, Aurora B, and EDU at 200 × magnification (α-Actinin, green; Ki67, pH3, Aurora B, and EDU, red; DAPI, blue. Scale bars, 200 μm). **(C,D)** Protein expression levels of PCNA as determined by Western blotting **(C)** in heart tissue from P0 and P7 rats and the corresponding densitometric analyses **(D)**. GAPDH was detected as the loading control. **(E,F)** Quantification of m6A in total RNA in heart tissue **(E)** and NRCMs **(F)** from P0 and P7 rats. *N* > 3 per group. The results are expressed as means ± SEMs (NS indicates not significant, *P < 0.05, **P < 0.01, ***P < 0.001, compared to the control group).

First, we assessed proliferation markers in heart tissue from P0 and P7 rats. The positive cells of Ki67, pH3, Aurora B, and EDU were significantly increased in P0, with rare proliferation cells in P7 (Figures 1A,B). The expression of PCNA protein was also decreased in P7 (Figures 1C,D). Second, we quantified the m6A level in heart tissue and NRCMs from the P0 and P7 rats. The same downregulating trend in tissue and cells was found in P7 and P0 (Figures 1E,F). It is conceivable, therefore, that m6A modification in RNA may be a key factor in cardiomyocyte regeneration.

### METTL3 Is Downregulated in P7 Relative to P0 Rats

**Figure 2:**
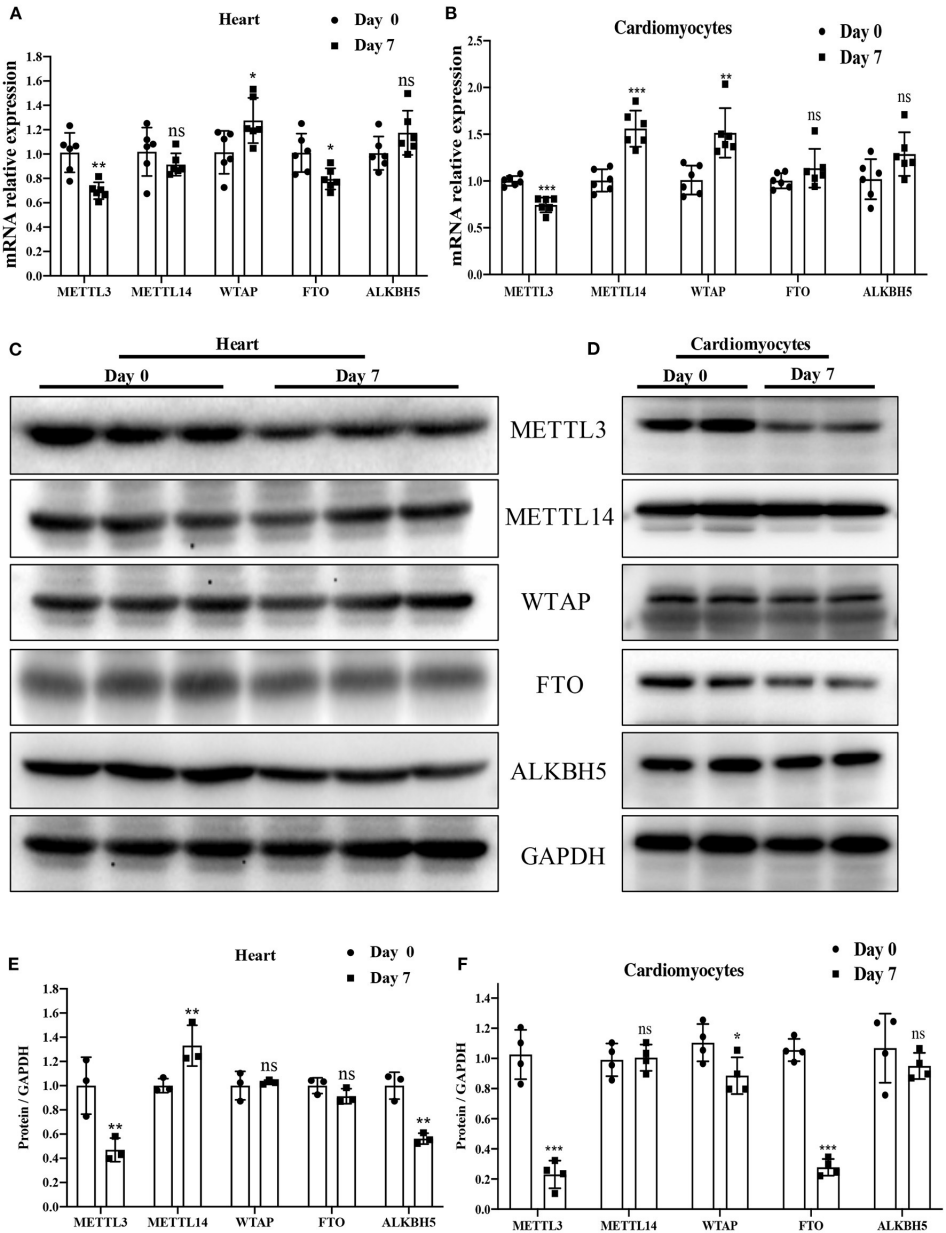
METTL3 was downregulated in P7 rat heart compared to P0. **(A,B)** mRNA expression level of the major enzymes: METTL3, METTL14, WTAP, FTO, and ALKBH5 in P0 and P7 rat heart **(A)** or cardiomyocytes **(B)** determined by the qPCR method. **(C–F)** Protein expression levels of the major enzymes METTL3, METTL14, WTAP, FTO, and ALKBH5 in P0 and P7 rat heart **(C)** or cardiomyocytes **(E)**, as determined by Western blotting and the corresponding densitometric analyses **(D,F)**. GAPDH was detected as the loading control. *N* > 3 per group. The results are expressed as means ± SEMs (NS indicates not significant, *P < 0.05, **P < 0.01, ***P < 0.001, compared to the control group).

We conducted qRT-PCR to examine the mRNA expression level of the major enzymes METTL3, METTL14, WTAP, FTO, and ALKBH5 in P0 and P7 rat heart or cardiomyocytes. Interestingly, mRNA levels of METTL3, the key methyltransferase regulating m6A modification, were significantly downregulated in P7 relative to P0 (Figures 2A,B). A similar decreasing trend in METTL3 was also found in the results of protein expression determined by Western blotting (Figures 2C–F). The downregulation of METTL3 is most relevant to the lower m6A modification in P7 than in P0, while several changes, such as ALKBH5 and FTO in tissue and METTL14, and ALKBH5 in cardiomyocytes, betrayed the change in m6A modification. These results likely indicate that METTL3 is the key regulator of m6A modification in cardiomyocyte proliferation.

### Overview of the m6A Methylation Map in Heart Tissues From P0 and P7 Rats

**Figure 3:**
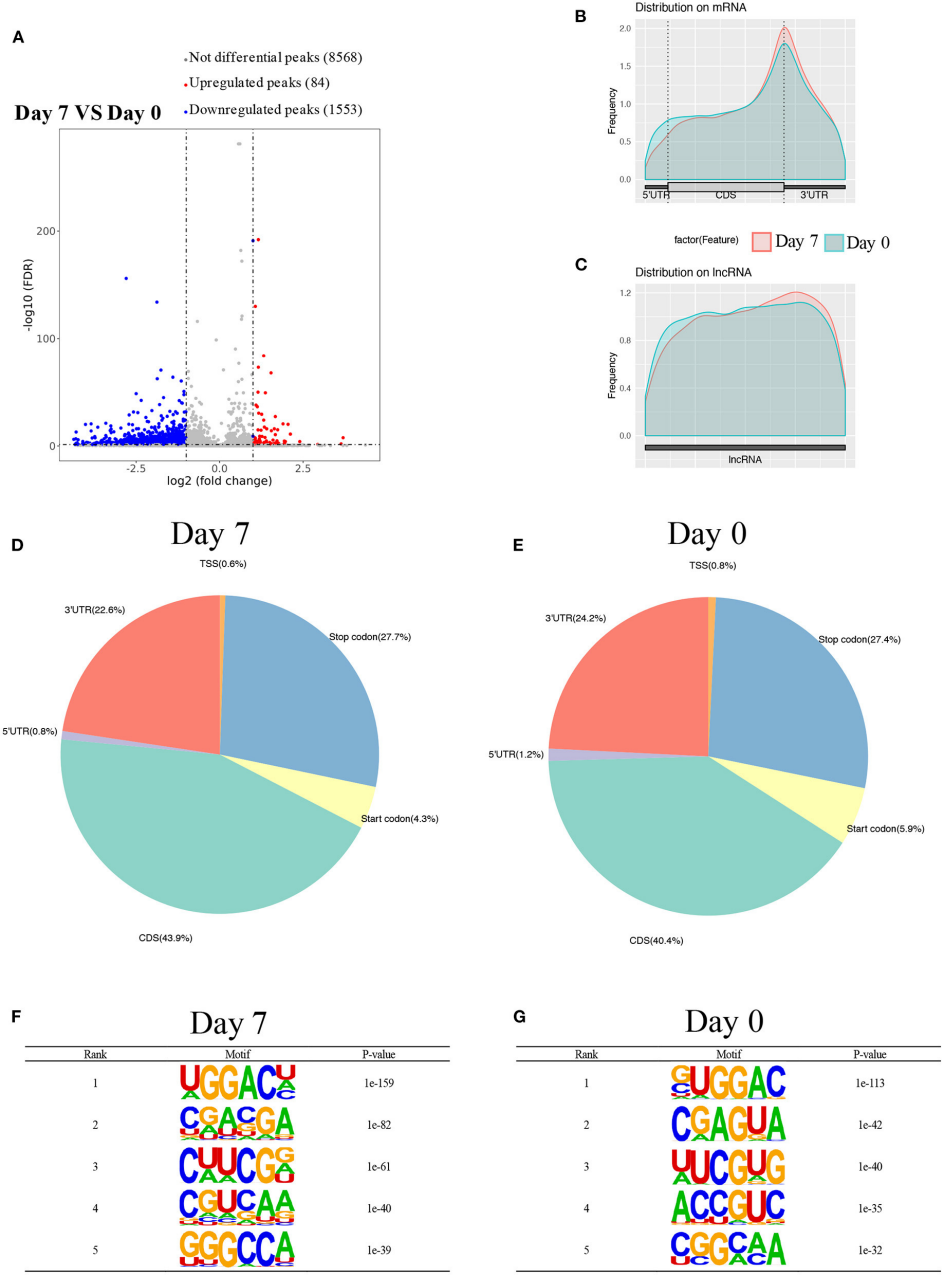
Overview of m6A methylation map in heart tissues from P0 and P7 rats. **(A)** Genome-wide profiling of m6A-modified mRNA and lncRNA in the heart from P0 and P7 rats. **(B,C)** Enriched m6A peaks in the coding sequence near the stop codon on mRNA and the distribution on lncRNA in the heart from P0 **(B)** and P7 **(C)** rats, and their statistical analyses **(D,E)**, respectively. **(F,G)** The top five m6A motifs of the m6A peaks in the heart from P0 **(F)** and P7 **(G)** rats.

Using MeRIP-seq, we performed genome-wide profiling of methylation-modified mRNA and lncRNA in the heart of P0 and P7 rats. As shown in Figure 3A, the P7 heart had 84 significantly upregulated methylation peaks relative to the P0 heart, and 1,553 were downregulated (fold changes ≥2). The top 20 altered methylation peaks are listed in Table 1, and the significant methylation peaks in the lncRNA are listed in Table 2. Figures 3B,C show that methylation peaks in both P0 and P7 were similarly enriched in the coding sequence (CDS) near the stop codon in mRNA and the distribution in lncRNA. In detail, methylation peaks in P7 heart tissues showed a different pattern from peaks in P0 heart tissues with a relatively increasing number of methylation peaks in the CDS (43.9 vs. 40.4%) and a relative decrease in the start codon (4.3 vs. 5.9%), the 5' untranslated region (5′UTR) (0.8 vs. 1.2%), and the 3′ untranslated region (3′UTR) (22.6 vs. 24.2%) (Figures 3D,E). In addition, the methylation peaks in P7 and P0 were characterized by the classic GGAC motif, and the top five m6A motifs are shown in Figures 3F,G. We analyzed the distribution of methylation peaks per mRNA or gene (Supplementary Figures 1A,B) and found that the majority of mRNAs or genes had a methylation peak (the mRNAs had 331 upregulated peaks and 1,971 downregulated peaks; the genes had 337 upregulated peaks and 2,002 downregulated peaks). After mapping to rat chromosomes, variant m6A peaks were found in all chromosomes except chrY, and they were particularly evident in chr1, chr2, chr12, and chr14 (Supplementary Figure 1C).

**Table 1:** Top 20 altered m6A peaks between the genome-wide profiling of m6A-modified mRNA in the heart from P0 and P7 rats.

**Chromosome**	**Peak start**	**Peak end**	**Gene name**	**Regulation**	**Fold change**	**Peak region**	***P*-value**
MT	5649	5887	Mt-co1	up	1.4804133	CDS	0
chr3	63567758	63568346	AABR07052585.2	up	1.52414483	CDS	1E-285
chr16	10880368	10881087	Ldb3	up	2.23457428	3′UTR	1E-195
chr6	4520065	4520604	Slc8a1	up	2	CDS	1E-194
chr14	114559830	114560250	Sptbn1	up	1.55401454	CDS	1E-185
chr1	225198523	225199634	Ahnak	up	1.58776786	CDS	1E-175
MT	11027	11537	Mt-nd4	down	0.14358729	CDS	1E-159
chr19	55710454	55712910	Ankrd11	down	0.27168372	CDS	1E-137
chr20	37888582	37889089	Gja1	up	2.09943337	3′UTR	1E-133
MT	13542	13687	Mt-nd6	up	1.59328019	CDS	1E-124
chr2	52189976	52190395	Nnt	up	1.57680035	3′UTR	1E-121
chr7	117231336	117237297	Plec	down	0.62937859	CDS	1E-119
chr1	225199782	225201826	Ahnak	up	1.38799272	CDS	5.01187E-94
chr1	255372256	255372704	Ppp1r3c	up	2.4966611	3′UTR	1.58489E-87
chr3	13841164	13842554	Hspa5	up	1.49174403	CDS	1.99526E-80
chr3	117939062	117939745	Eid1	up	2.23457428	3′UTR	1.25893E-76
chr11	42945083	42946693	Crybg3	down	0.52195596	CDS	1.25893E-72
chr6	10704350	10704798	Socs5	up	2.90794503	3′UTR	3.16228E-71
chr10	11283012	11283253	Srl	up	1.9574833	3′UTR	1.25893E-69
chr5	167659493	167660152	Rere	down	0.37892914	CDS	3.98107E-67

*MT, mitochondrial DNA; 3′UTR, 3′ untranslated region; CDS, coding sequence; exon: expressed region*.

**Table 2:** Significant m6A peaks among lncRNA in the heart from P0 and P7 rats.

**Chromosome**	**Peak start**	**Peak end**	**Gene name**	**Regulation**	**Fold change**	**gene_biotype**	***P*-value**
chr20	4436748	4438703	AABR07044388.2	down	0.29524817	processed_transcript	7.9433E-74
chr1	41794310	41794761	AABR07001382.1	down	0.46976137	processed_transcript	7.9433E-26
chr20	21878721	21880715	Arid5b	down	0.56370021	processed_transcript	1.2589E-25
chr1	221158213	221158792	AC134224.3	up	1.03777954	lincRNA	1.2589E-23
chr1	41795269	41796349	AABR07001382.1	down	0.43226862	processed_transcript	1.9953E-21
chr12	11538398	11538968	Trrap	down	0.19614602	processed_transcript	1.2589E-11
chr14	60057609	60058128	AABR07015507.1	up	1.73989471	processed_transcript	1.7783E-06
chr8	65282854	65283121	AABR07070312.1	down	0.30145196	processed_transcript	2.4547E-06
chr16	36258624	36259072	AABR07025385.1	down	0.20447551	lincRNA	1.9055E-05
chr10	89168692	89168970	AABR07072184.3	down	0.52595089	antisense	2.6303E-05
chr6	98964482	98964752	AABR07064873.1	down	0.47963206	processed_transcript	6.7608E-05
X	71967172	71968516	AABR07039210.1	down	0.53849319	processed_transcript	8.9125E-05
chr6	99000864	99001434	AABR07064873.1	down	0.42044821	processed_transcript	0.00028184
chr8	119377867	119378197	AABR07073453.1	down	0.14660437	lincRNA	0.00032359
chr9	69899726	69899965	AC141169.2	down	0.03564887	lincRNA	0.00042658
chr13	80438873	80439233	Eef1aknmt	down	0.22687979	processed_transcript	0.00056234
chr2	41528906	41529086	LOC108349943	down	0.02303546	lincRNA	0.00095499
chr10	89181560	89181860	AABR07072184.2	down	0.28519093	processed_transcript	0.00165959
chr10	90932082	90932587	AABR07030514.1	down	0.38958229	antisense	0.00323594
chr1	187784719	187785770	Smg1	up	1.1352421	processed_transcript	0.0040738
chr8	13524076	13524401	AABR07069227.1	down	0.12762652	lincRNA	0.00776247
chr2	184693527	184693855	AABR07012065.1	down	0.03443453	lincRNA	0.01047129
X	71972301	71972810	AABR07039210.1	up	1.5822746	processed_transcript	0.01548817

### Differentially Methylated mRNAs Enriched in Important Signaling Pathways

**Figure 4:**
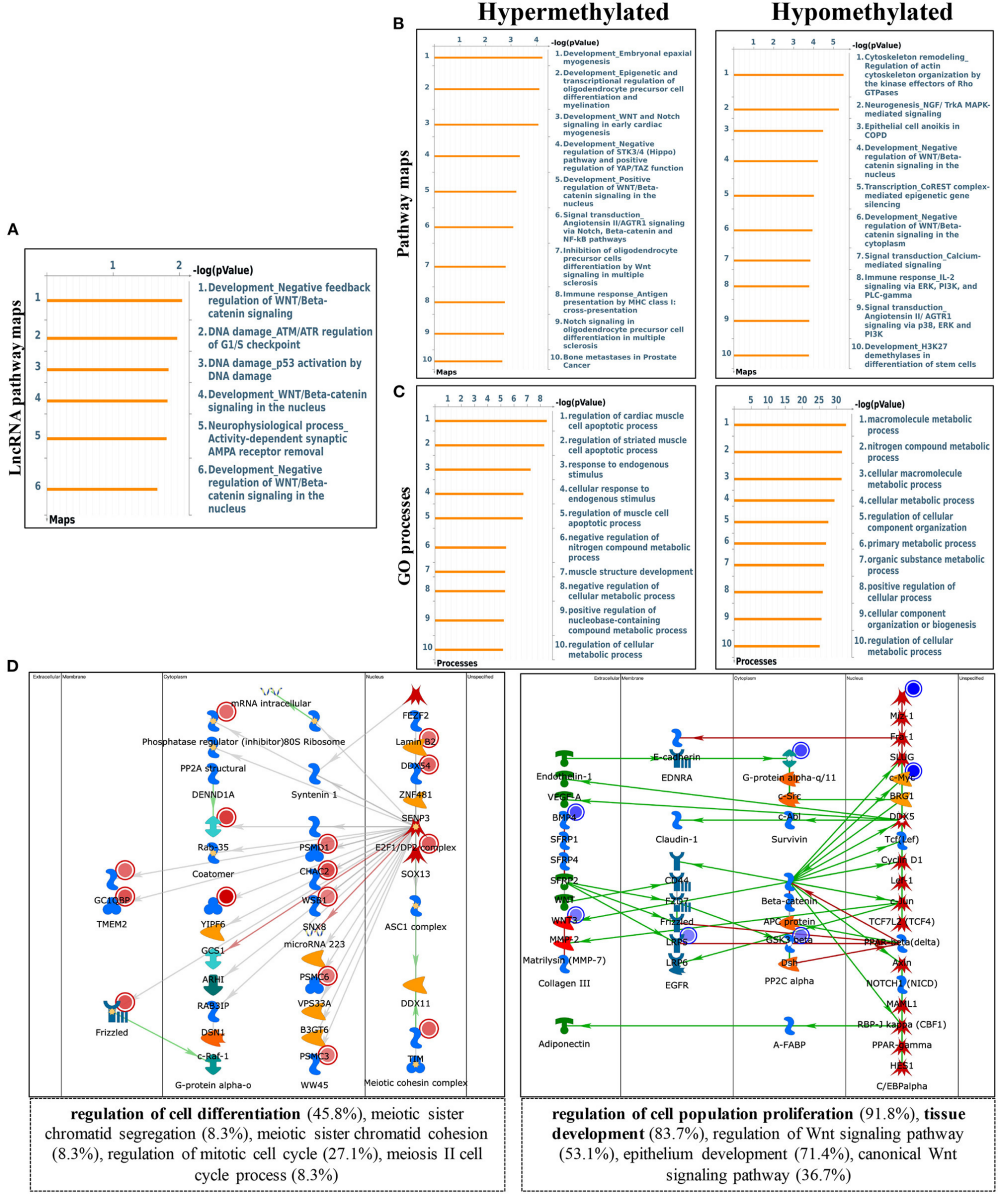
Pathway analysis of differentially m6A peaks. **(A)** Pathway Maps analysis of differentially methylated lncRNA. **(B)** Pathway Maps analysis of differentially methylated peaks. Left panel represents hypermethylated genes and right panel represents hypomethylated peaks. **(C)** Gene Ontology (GO) process analysis of differentially methylated peaks. Left panel represents hypermethylated peaks and right panel represents hypomethylated peaks. **(D)** Top scored networks analysis of differentially methylated peaks. Left panel represents hypermethylated genes and right panel represents hypomethylated peaks.

To explore the biological significance of methylation modification in cardiomyocyte regeneration, we performed GO analyses and KEGG pathway analyses for the altered methylation peaks. GO enrichment analyses revealed the top 20 enrichments (Supplementary Figure 1D). In addition, pathway-enrichment analyses showed that methylation modifications in P0 and P7 heart tissues were significantly associated with focal adhesion, the Notch signaling pathway, the mTOR signaling pathway, and pathways in cancer (Supplementary Figure 1C). By using Metacore system, pathway maps analysis results showed that dys-methylated lncRNA were significantly enriched in development negative feedback regulation of WNT/Beta-catenin signaling and DNA damage ATM/ATR regulation of G1/S checkpoint (Figure 4A). In the pathway map analysis of mRNA, the hypermethylated peaks were strongly associated with development embryonal epaxial myogenesis, and development epigenetic and transcriptional regulation of oligodendrocyte precursor cell differentiation and myelination. Besides, the most relevant biological pathways of hypomethylated peaks were cytoskeleton remodeling and neurogenesis NGF/TrkA MAPK-mediated signaling (Figure 4B). In addition, GO process results showed that the hypermethylated peaks were significantly enriched in the regulation of cardiac muscle cell apoptotic process and regulation of striated muscle cell apoptotic process, while the hypomethylated peaks were enriched in macromolecule metabolic process and nitrogen compound metabolic process (Figure 4C). Furthermore, the top-scored network analyses of hypermethylated peaks about cardiomyocyte regeneration were found to be involved in regulation of cell differentiation (45.8%), meiotic sister chromatid segregation (8.3%), meiotic sister chromatid cohesion (8.3%), regulation of mitotic cell cycle (27.1%), meiosis II cell cycle process (8.3%) (Figure 4D and Supplementary Table 2). The top-scored networks of hypomethylated peaks about heart regeneration were enriched in regulation of cell population proliferation (91.8%), tissue development (83.7%), regulation of Wnt signaling pathway (53.1%), epithelium development (71.4%), canonical Wnt signaling pathway (36.7%) (Figure 4D and Supplementary Table 2).

### Overview of Transcriptome Profiles and Conjoint Analyses of MeRIP-Seq and RNA-Seq Data

**Figure 5:**
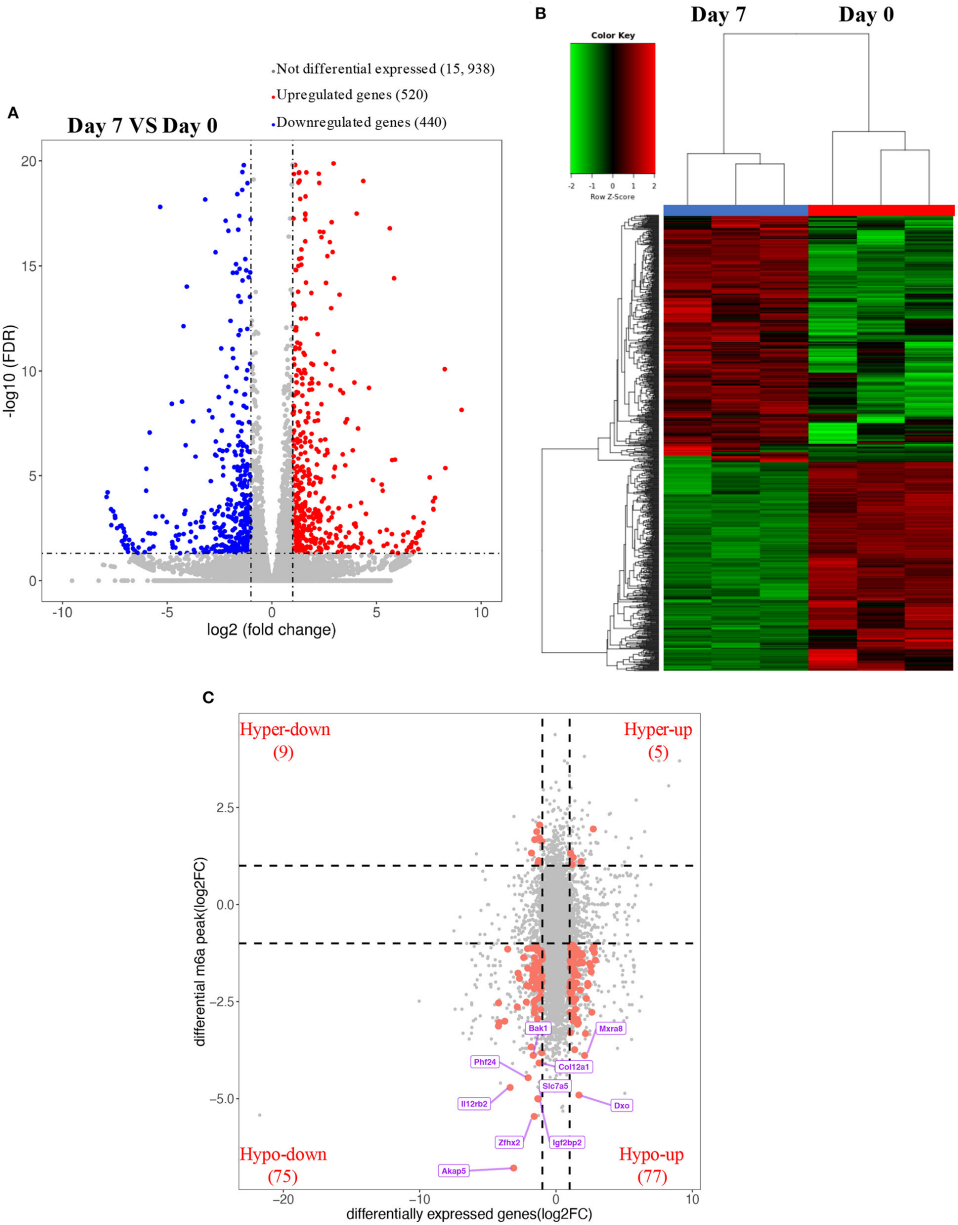
Overview of transcriptome profiles and conjoint analyses of MeRIP-seq and RNA-seq data. **(A,B)** Upregulated and downregulate genes in rat hearts compared between P0 with P7 shown in RNA-seq. **(C)** m6A peaks in RNA transcripts identified after conjoint analyses of MeRIP-seq and RNA-seq data. MeRIP-seq, m6A RNA immunoprecipitation sequencing.

**Figure 6:**
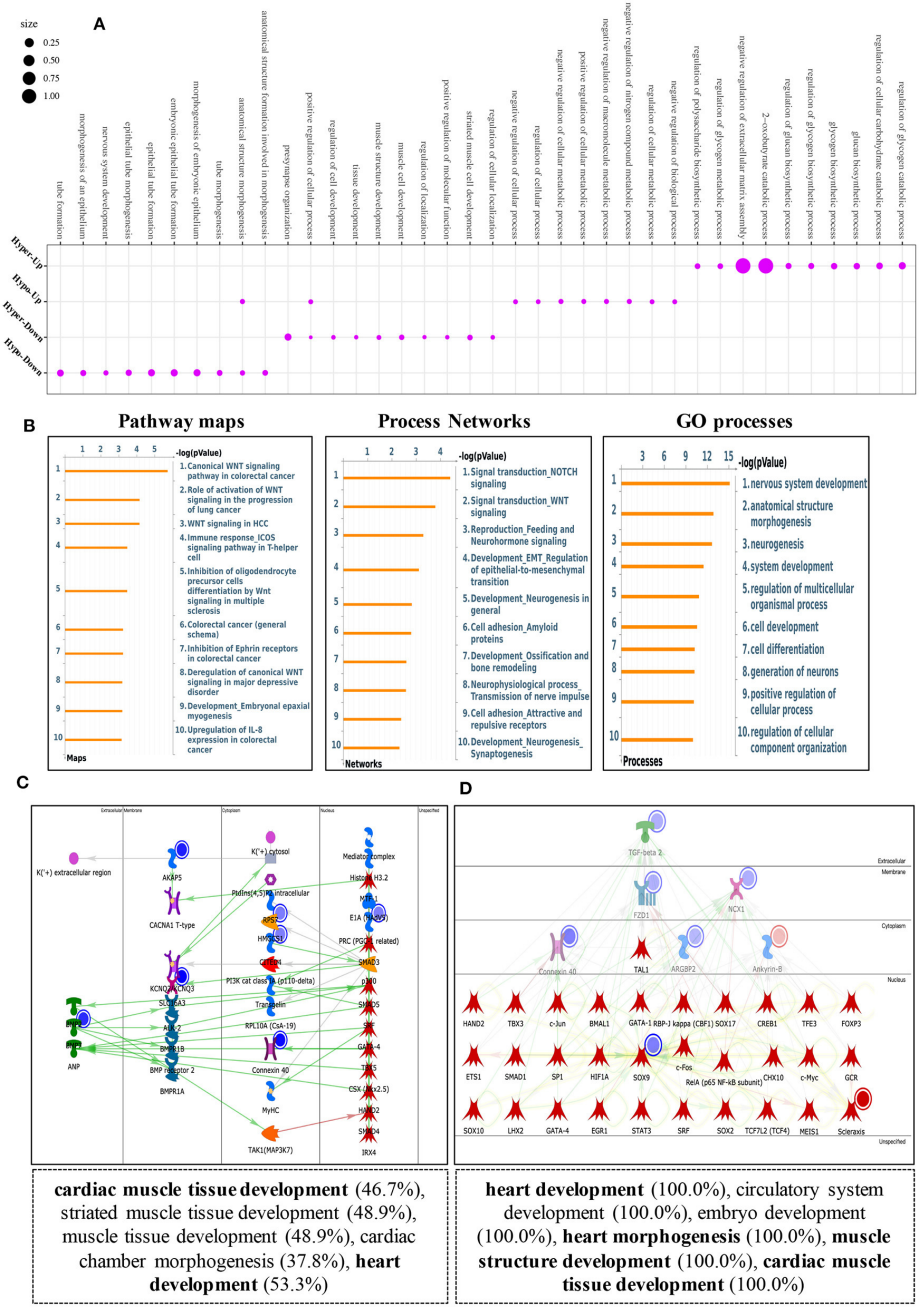
Key pathway analysis of differentially m6A-modifed genes in MeRIP-seq and RNA-seq data. **(A)** Bubble diagram of GO biological process categories enriched for DEGs with m6A hyper- or hypo-methylated. **(B)** Pathway maps analysis (left), Process networks analysis (middle), GO process analysis (right) of differentially m6A-modifed genes. **(C)** Top scored networks analysis of m6A-modified genes. **(D)** Key transcription factors and target genes network analysis of m6A-modifed genes.

Using RNA-seq, we found the transcriptome profiles of genes altered between P0 and P7. In P7 heart tissues, 440 significantly downregulated genes and 520 significantly upregulated genes relative to P0 are shown in Figures 5A,B. The top 20 differentially expressed genes are listed in Table 3, and significantly expressed lncRNAs are listed in Table 4. We performed conjoint analyses of MeRIP-seq and RNA-seq data and identified only 14 hypermethylated m6A peaks in RNA transcripts that were significantly upregulated (5, hyper-up) and downregulated (9, hyper-down), and 152 hypomethylated m6A peaks in RNA transcripts that were significantly upregulated (77, hypo-up) and downregulated (75, hypo-down) (Figure 5C). The GO analyses of processes associated with the four gene sets showed detail in Figure 6A, which apparently linked to numerous functional processed and pathways. Then the 166 significant changes in both methylation modification and RNA expression levels were subjected to pathways maps, process networks, and GO processes (Figure 6B). Interestingly, the signal transduction NOTCH signaling and Wnt signaling were enriched in process networks. The top 15 GO and KEGG pathways that were up-or downregulated in both methylation modification and RNA expression genes are shown in Supplementary Figure 2. Moreover, the top-scored network analyses indicated that methylation-modified genes were significantly enriched in regulation of cardiac muscle tissue development (46.7%), striated muscle tissue development (48.9%), muscle tissue development (48.9%), cardiac chamber morphogenesis (37.8%), heart development (53.3%) (Figure 6C and Supplementary Table 3). After analyzed the key transcription factors and target genes in methylation-modified genes, we found eight genes (TGF-beta 2, FZD1, NCX1, Connexin 40, ARGBP2, Ankyrin-B, SOX9, and Scleraxis) are most enriched in heart development (100.0%), circulatory system development (100.0%), embryo development (100.0%), heart morphogenesis (100.0%), muscle structure development (100.0%), cardiac muscle tissue development (100.0%) (Figure 6D). These observations indicate that genes with m6A modification may play important role in regulating cardiomyocyte regeneration.

**Table 3:** Top 20 differentially expressed genes in rat hearts comparing the P0 with P7 after RNA-seq.

**Gene name**	**Regulation**	**Fold change**	***P*-value**
Cmya5	up	5.9694651	4.69E-192
Slc16a12	down	0.05400898	4.53E-101
Pdk4	up	5.21115443	1.62E-99
Myh14	up	5.77786592	6.42E-96
Tcp11l2	up	5.89883045	8.52E-83
Coq8a	up	5.47823692	2.03E-80
Spta1	up	18.9695639	5.84E-78
Ezr	down	0.29291865	1.89E-72
Slc8a1	down	0.31828392	1.04E-71
Pde4dip	up	3.30849372	7.48E-64
Myl4	down	0.13470013	1.68E-60
Sfrp1	down	0.14520323	1.30E-57
Tnni1	down	0.33747257	6.54E-54
Itga7	up	6.69544417	1.89E-53
Tgm2	down	0.35943868	2.87E-51
Fignl1	down	0.0903453	2.96E-48
Gyg1	down	0.25541332	4.50E-48
Acss1	up	11.1592641	1.45E-46
C1qtnf9	up	13.7984422	4.44E-46
Myom2	up	11.5950342	1.15E-44

**Table 4:** Significantly expressed lncRNAs in rat hearts comparing P0 with P7 after RNA-seq.

**Gene name**	**Regulation**	**Fold change**	***P*-value**
AABR07044388.2	up	2.67376633	8.00E-49
AABR07072236.1	up	2.12257436	3.60E-06
AABR07060487.1	up	210.276458	1.87E-05
AABR07001555.1	up	4.92616845	3.66E-05
AABR07050487.1	up	8.61721842	0.000229068
AABR07007026.1	up	4.84495817	0.000241822
AABR07010868.1	up	134.04159	0.000467942
AABR07049292.1	up	78.1693608	0.000511441
AABR07063682.1	up	135.24403	0.000562795
AABR07052523.2	up	2.62925418	0.000635361
AABR07031489.1	up	8.53172104	0.000822179
AABR07035722.1	up	60.9739333	0.002399276
AABR07058805.1	up	94.0379143	0.002748284
AABR07035868.1	up	54.3294555	0.005296683
Bves	down	0.27545246	1.84E-17
AABR07055191.1	down	0.00522842	2.51E-05
AABR07021456.1	down	0.05523855	3.47E-05
AABR07064878.1	down	0.00552921	4.50E-05
AABR07049799.1	down	0.10319418	0.000184943
AC119762.7	down	0.16163214	0.000197815
AABR07030834.1	down	0.1507684	0.000212773
AABR07026924.1	down	0.01677124	0.000416586
AABR07031234.1	down	0.0077618	0.000703548
AABR07045400.1	down	0.0099293	0.001797653
AABR07065531.5	down	0.29224304	0.00198907
AC119007.3	down	0.00801545	0.002591027
LOC102551356	down	0.14495554	0.003067112
AABR07021888.1	down	0.01004747	0.003280718
AABR07041096.1	down	0.42367893	0.004457441
AABR07069218.2	down	0.20815401	0.00462579
AABR07040629.1	down	0.35360747	0.005334635
AABR07049695.4	down	0.06052745	0.006198736

### Screening and Validating the Most Relevant Genes for Cardiomyocyte Regeneration by Combining the GEO Datasets

**Figure 7:**
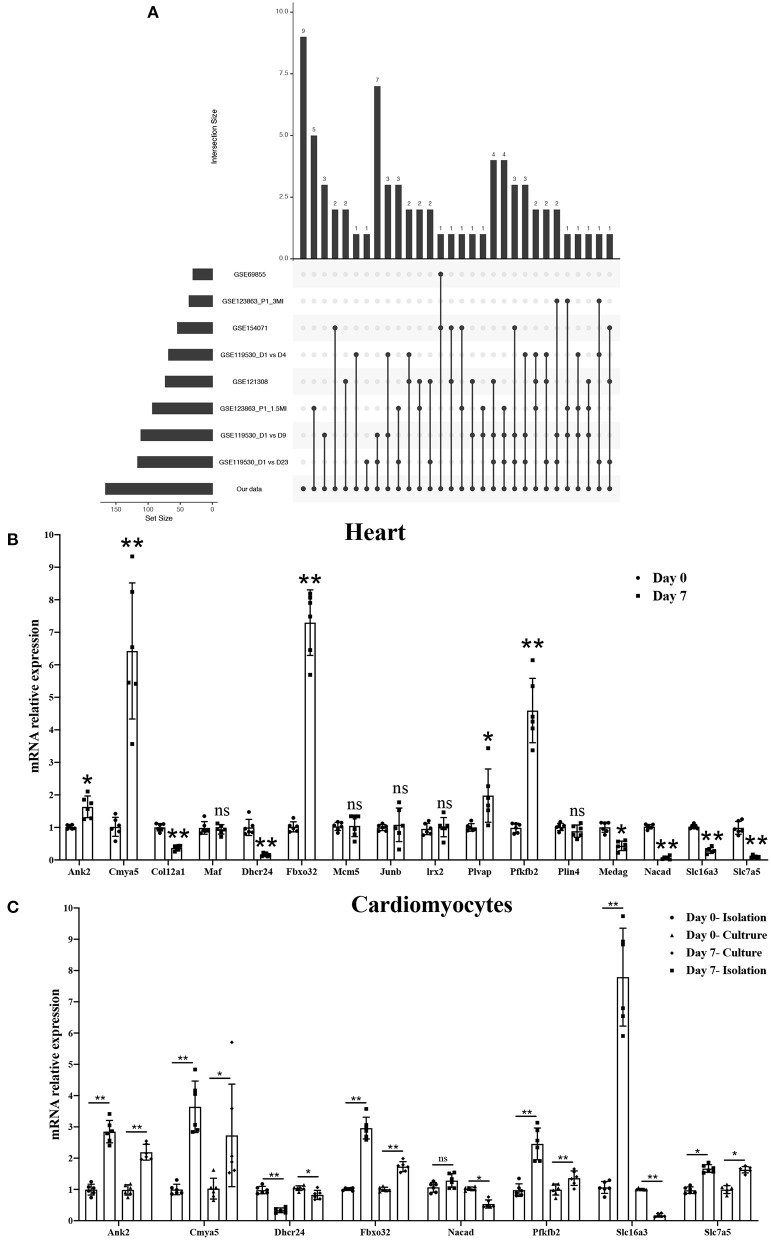
Screening and validating the most relevant genes for cardiomyocyte regeneration by combining GEO datasets. **(A)** Hub genes showing significant differences in three GEO datasets associated with cardiomyocyte regeneration and our study. **(B)** mRNA expression level of 16 hub genes in P0 and P7 rat heart tissues determined by the qPCR method. **(C)** mRNA expression level of eight genes in isolated and cultured cardiomyocytes as determined by the qPCR method. *P < 0.05, **P < 0.01, compared to the Day 0 group.

To further validate the most relevant gene correlations with cardiomyocyte regeneration, we examined 166 significant genes in GEO datasets, including GSE154071, GSE121308, GSE69855, GSE119530, and GSE123863, which have relevance for the study of cardiomyocyte regeneration. Ultimately, 16 hub genes were identified that showed significant differences between the five GEO datasets and our study (Figure 7A). qRT-PCR was used to validate their expression in P0 and P7 heart tissues and cardiomyocytes. The results show the same trend among eight genes in heart tissues, including increased expression of Ank2, Cmya5, Fbxo32, and Pfkfb2 and decreased expression of Dhcr24, Nacad, Slc16a3, and Slc7a5 (Figure 7B). Interestingly, Ank2, Cmya5, Fbxo32, Pfkfb2, and Dhcr24 showed synchronous expression in isolated and cultured cardiomyocytes, but only cultured cardiomyocytes showed the same trend with Nacad and Slc16a3. Moreover, the expression of Slc7a5 in cultured or isolated cardiomyocytes was significantly contrary (Figures 7C,D). Besides, the detailed data visualization of m6A modification in these eight genes were shown in Supplementary Figure 3.

### The Functional Link of METTL3 Expression to Transcript Stability of Target Genes

**Figure 8:**
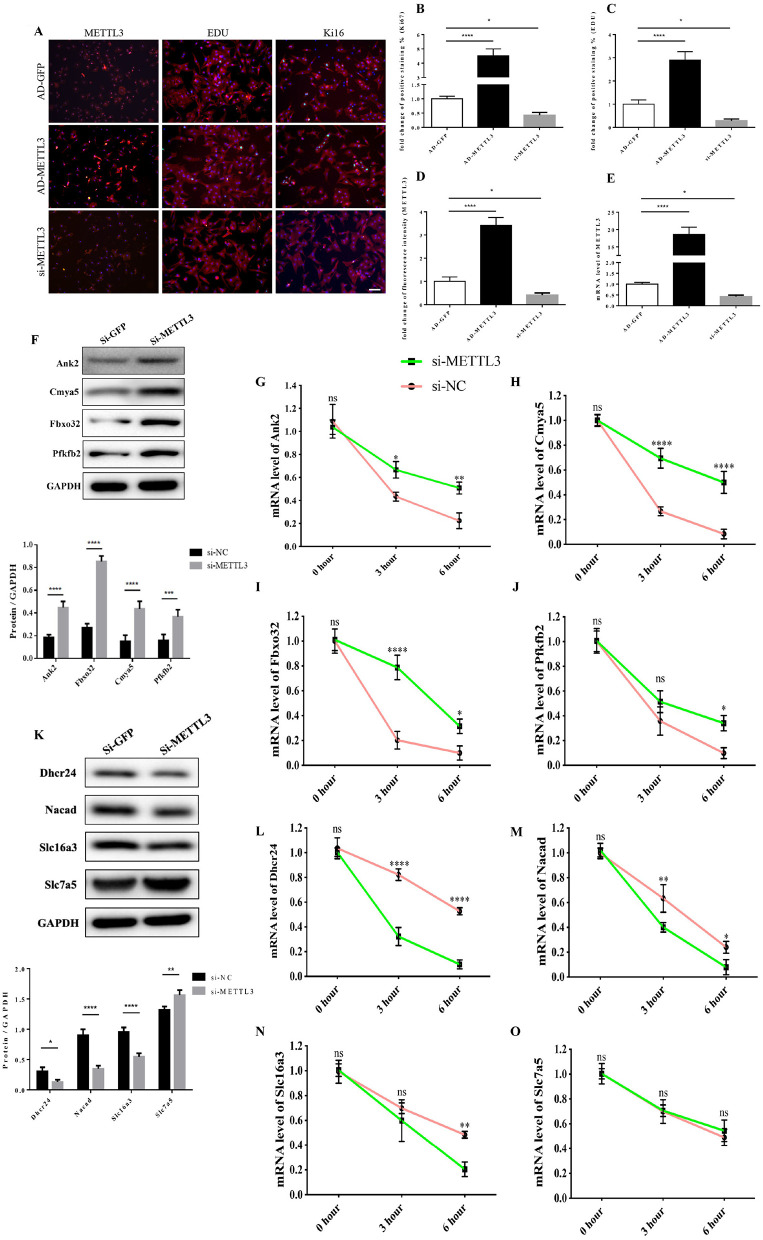
The functional link of enhanced METTL3 expression to transcript stability of target genes. **(A–D)** Representative immunofluorescence images of NRCMs from P0 rat hearts labeled with METTL3, EDU, and Ki67 (METTL3, or α-actinin, red; EDU, or Ki67, green; DAPI, blue. Scale bars, 50 μm) **(A)** and their corresponding quantitative analysis **(B–D)**. **(E)** mRNA expression level of METTL3 of NRCMs from P0 rat hearts transfected with AD-GFP, AD-METTL3, or si-METTL3 determined by the qPCR method. **(F)** Protein expression levels of Pfkfb2, Ank2, Cmya5, and Fbxo32 in P0 NRCMs transfected with si-NC or si-METTL3 (up) and the corresponding densitometric analysis (down). **(G–J)** mRNA expression level of Ank2 **(G)**, Cmya5 **(H)**, Fbxo32 **(I)**, and Pfkfb2 **(J)** in P0 NRCMs transfected with si-NC or si-METTL3 after treated with 20μg/ml Actinomycin D for 0, 3 or 6 hours. **(K)** Protein expression levels of Dhcr24, Nacad, Slc16a3, and Slc7a5 in P0 NRCMs transfected with si-NC or si-METTL3 (up) and the corresponding densitometric analysis (down). **(L–O)** mRNA expression level of Dhcr24 **(L)**, Nacad **(M)**, Slc16a3 **(N)**, and Slc7a5 **(O)** in P0 NRCMs transfected with si-NC or si-METTL3 after treated with 20 μg/ml Actinomycin D for 0, 3 or 6 h. GAPDH was detected as the loading control. *P < 0.05, **P < 0.01, ****P < 0.001, *****P < 0.0001, compared to the si-NC group.

First, in order to further validate the function of METTL3 in cardiomyocytes. We conducted a cell proliferation assay in cardiomyocytes by interference or overexpression of METTL3. Compared with the control group, the number of Ki67 and EDU positive cells in P0 cardiomyocytes overexpressed with METTL3 was significantly increased, while rare proliferating cells were found in P0 cardiomyocytes knocked out with METTL3 (Figures 8A–E and Supplementary Figure 4). Then, we performed the transcript stability assays post-METTL3 silencing to further explore the functional link of changes in methylation status to transcript stability or protein abundance of identified targets. The actinomycin D-RNA stability assays showed that the identified genes, including Dhcr24, Nacad, and Slc16a3, displayed a consistent and relative higher rate of decay of mRNA levels in the absence of METTL3 than the control group (Figures 8F–O), while Pfkfb2, Ank2, Cmya5, and Fbxo32 showed a relative stable rate of mRNA decay in both two groups, indicating that the enhanced METTL3 expression and methylation levels contributed to reduced mRNA decay and increased transcriptional stability of the identified genes.

## Discussion

The morbidity and mortality associated with cardiovascular diseases worldwide remain high even as living standards improve and medical conditions become ameliorated (23). The loss of cardiomyocytes is thought to be a major cause of cardiac dysfunction, eventually resulting in local myocardial necrosis and irreversible fibrosis. Epigenetics, including DNA methylation, chromatin remodeling, and histone modifications, has gained considerable attention due to its role in the regulation of cardiomyocyte proliferation (24). M6A methylation is a reversible and heritable chemical modification in epigenetic regulation that can lead to a variety of common pathological reactions, including ischemia, inflammation, and tumorigenesis.

Recent studies have shown that m6A epigenetic regulation is closely related to a variety of cardiovascular diseases (18, 19) and may become a new clinical therapeutic target. This indicates the major role and importance of m6A regulation in eukaryotic genomes that influence the physiological and pathological processes in an organism, particularly in the process of myocardial development. Studies have confirmed that the conserved sequences contained in human and mouse m6A are highly homologous, and the mRNA levels of both are regulated dynamically from embryonic to adult stages (25, 26). Compared with adults, tissues during embryonic development have higher m6A levels, suggesting that m6A plays an irreplaceable role in growth and development. It has been reported that Mettl3 and Mettl14 can negatively regulate the stability of RNAs by regulating m6A levels, thereby maintaining or even improving the self-renewal ability of embryonic stem cells (mESCs) (27). Besides, FTO has also been found to play a crucial role in the early development of the human central nervous system, and cardiovascular system (28). In recent years, most studies showed there are interactions between miRNAs with m6A in the regulation of messenger RNA (mRNA) stability (29). Even the regulation between themselves is present in several studies (30, 31). Thus, it would be interesting and valuable to explore the function between miRNAs and m6A in cardiomyocyte proliferation. We summarized the miRNAs and targets involved in cardiomyocyte proliferation. Interestingly, these targets showed quite a difference in m6A modification and mRNA expression. The vast majority of these targets change consistent with the regulation of miRNAs in cardiomyocyte proliferation. While, the m6A levels of these targets seem quite different in P7 compared to P0 (Supplementary Table 4). These differences between m6A levels and the expression of targets may be caused by the m6A reader (YTHDF1, YTHDF2, and YTHDF3) which plays different roles in the fate of m6A modified mRNA. However, this hypothesis still needs follow-up research to confirm it.

This study was the first to reveal the m6A landscape of the heart in the neonatal rats at P0 and P7. First, we found that the total RNA m6A level was dramatically decreased in the heart tissue and NRCMs in P7 compared to P0. The downregulating trend of METTL3 was detected by qRT-PCR and Western blotting. Thus, the decrease in METTL3 is the most relevant to the downregulation of m6A modification in P10, which indicates that m6A modification and its regulator METTL3 may be key factors in cardiomyocyte regeneration. We performed genome-wide profiling of m6A-modified mRNA and lncRNA in hearts from P0 and P7 rats using m6A-RIP-seq. First, 1,637 m6A peaks were significantly differentiated, with 84 being upregulated and 1,553 downregulated. In addition, GO analyses were performed, and the most relevant were DNA synthesis and angiogenesis. As previously reported, the microscopic presence of mitoses contributes to cardiac regeneration, as supported by the fact that most human cardiomyocytes seem to be mononucleated (32). Furthermore, the lack of an apparent regeneration response or neovascular response observed in adult mammal hearts suggests that angiogenesis may be particularly vital for cardiac regeneration (33, 34). A range of molecular circuitries are essential to the proliferation of heart muscle cells and are required in the formation of the ventricular trabeculation and chamber, as well as in the maintenance of cardiac function in their maturation. However, few molecular mechanisms are fully known due to the complexity underlying cardiac regeneration (35). The KEGG pathway analyses showed that the Notch, mTOR, and Wnt signaling pathways were significantly different between P0 and P7 heart tissues. Grego-Bessa (36) and Chen (37) showed that Notch1 activity gradually becomes concentrated from the ventricular trabecular endocardium to the base and activates Notch-dependent genes, such as BMP10, which is responsible for heart regeneration and maturation. Wnt/β-catenin signaling can activate mTOR signaling or other pathways, and it partners with them to orchestrate cardiac regeneration (38). The activation of the mTOR signaling pathways is reported to have a cardioprotective effect against MI or other cardiac injury-induced cardiac dysfunction or heart failure via the promotion of autophagy, which is important for cardiac energy homeostasis (39–41). However, several studies have shown that these pathways are important for the state of the physiological and pathological processes of the heart, but their specific role in cardiac regeneration is less well-known.

Finally, conjoint analyses of MeRIP-seq and RNA-seq data were performed to confirm m6A-modified RNA transcripts, which were significantly different in their m6A level and expression. We discovered 166 genes with significant differences in the levels of both m6A modification and RNA expression. GO and KEGG pathway analyses of these 166 genes showed enrichment of dipeptide transport, positive regulation of DNA damage checkpoint and spermine transport, and mismatch repair, homologous recombination, and DNA replication. Furthermore, in relation to GEO datasets, which were a major focus in the study of cardiomyocytes regeneration, 8 (Ank2, Cmya5, Dhcr24, Fbxo32, Nacad, Pfkfb2, Slc16a3, and Slc7a5) of 16 hub genes showed the same trend in the GEO dataset, RNA-seq data, and qRT-PCR on heart tissues and cardiomyocytes. Four (Ank2, Cmya5, Fbxo32, and Pfkfb2) of the eight hub genes were increased in P7 heart tissues. It has been reported that some of these genes play an independent role in heart disease. For example, although variants of Ank2 are associated with arrhythmia syndromes (42), sinus node disease (43), structural heart disease (44), and sudden cardiac death (45), their roles in cardiac proliferation are still unknown. Cmya5, encoded myospryn, functions as a negative regulator in skeletal muscle regeneration by inhibiting calcineurin-dependent transcriptional activity (46). A high expression of Fbxo32 has been found in muscle atrophy (47), while lower expression contributes to tumorigenesis in gastric cancer (48) and cervical neoplastic keratinocytes (49). Cellular metabolism in the heart is dynamic at different stages of cardiac myocytes. Glycolysis and fatty acid oxidation change from fetal to mature tissue (50). Pfkfb2 has been reported to play a special role in regulating glycolysis and proliferation in pancreatic cancer cells (51). In addition, four (Dhcr24, Nacad, Slc16a3, and Slc7a5) of eight hub genes were found to be decreased in P7 heart tissues. Dhcr24, which is involved in the final step of cholesterol synthesis via the Bloch pathway, plays an important role in multiple developmental anomalies (52). Interestingly, the expression of Nacad and Slc16a3 in P7 heart tissues and cultured cardiomyocytes was synchronous with the sequence data, but that of isolated cardiomyocytes was contrary. These differences between isolated cardiomyocytes and tissues may be caused by long-term isolation and relative hypoxia effects (53). As for Slc7a5, a glutamine transporter, its expression in P7 cardiomyocytes showed a significant increase. This may be glutamine is a usual supplement in isolation buffer and culture media. (54). Overall, the hub genes regulated by m6A modifications and the effects of cardiac regeneration may be considered to be crucial and independent. However, further study is necessary to uncover the precise mechanisms of the genes underlying cardiac regeneration. METTL3 and METTL14 can negatively regulate the stability of RNAs by regulating m6A levels, thereby maintaining or even improving the ability of embryonic stem cells for self-renewal (27). Further, FTO also plays a crucial role in the early development of the human central nervous system (28). Direct modulation of m6A modifications could become novel territory for the study of cardiac regeneration.

## Conclusion

**Figure 9:**
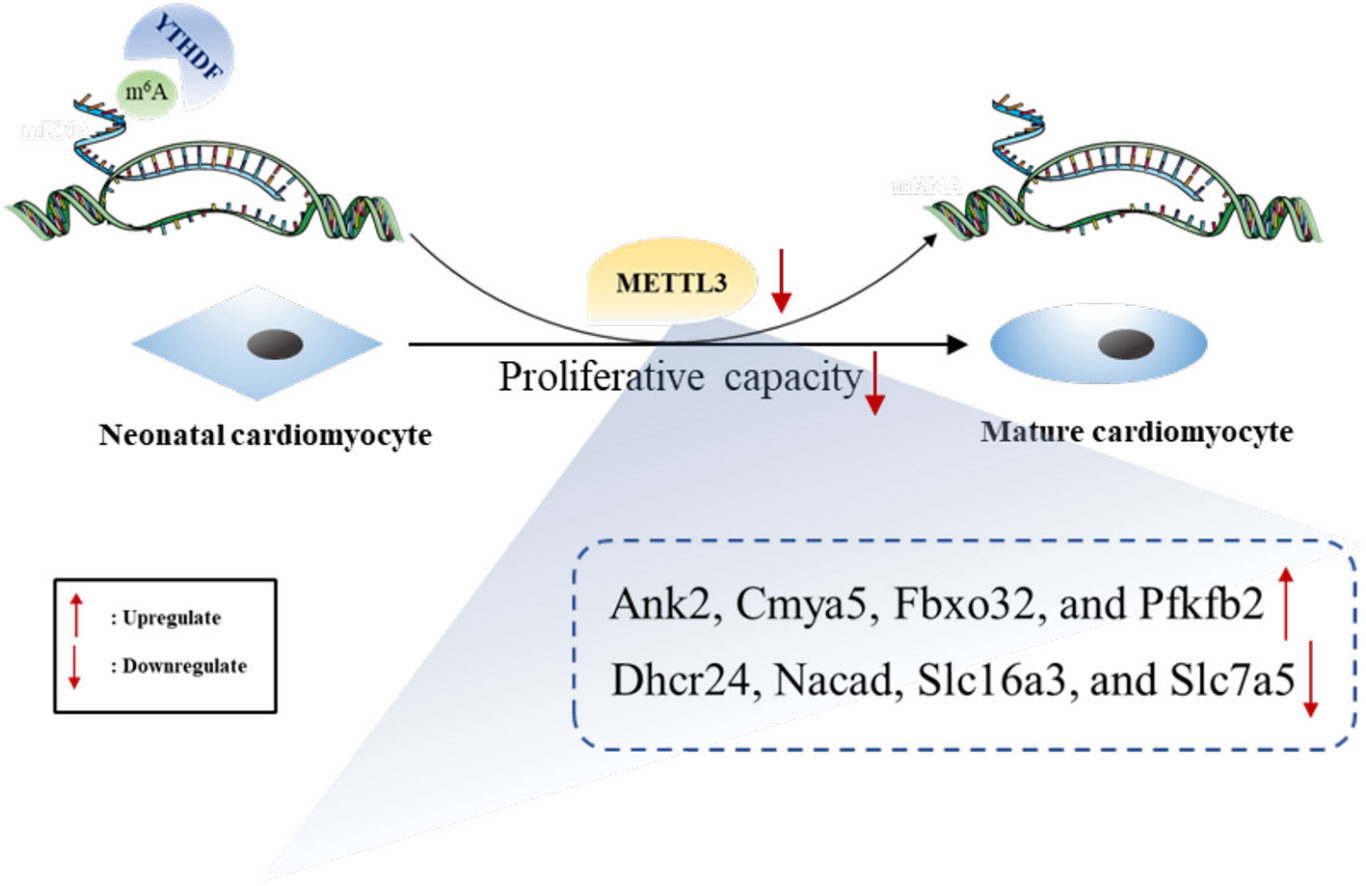
The summarized figure accounting for the potential role of METTL3 in neonatal cardiomyocyte proliferation.

The expression of both METTL3 and m6A in total RNA were significantly more downregulated in heart tissues from P7 than in those from P0 rats. We performed genome-wide analyses of m6A-modified transcripts and bioinformatics analyses to discover the potential functions of genes. Furthermore, conjoint analyses of m6A-RIP-seq, RNA-seq data, and GEO data generated eight potential hub genes with differentially expressed hypermethylated or hypomethylated m6A levels. Overall, increased METTL3 expression and the subsequent hypermethylated m6A levels may enhance the proliferative capacity of neonatal cardiomyocytes (Figure 9).

## Data Availability Statement

The datasets presented in this study can be found in online repositories. The names of the repository/repositories and accession number(s) can be found at: NCBI GEO; GSE162545.

## Ethics Statement

All procedures were approved by the Experimental Animal Care and Use Committee of Nanjing Medical University and conducted in accordance with the guide for the Care and Use of Laboratory Animals (NIH publication no. 85–23, revised 1996), under the approval number IACUC-1712010.

## Author Contributions

XK and JS designed the work and prepared the manuscript. CY, KZ, and JZ performed the experiments, conducted data analysis, and wrote the manuscript. XW and WS analyzed and interpreted the data. All authors discussed the results, read, and approved the final version of the manuscript for publication.

## Conflict of Interest

The authors declare that the research was conducted in the absence of any commercial or financial relationships that could be construed as a potential conflict of interest.

## Correction Note

A correction has been made to this article. Details can be found at: 10.3389/fcvm.2025.1662054.

## Supplementary Material

Supplementary Figure 1 Distribution and signaling pathways of m6A peaks and differentially expressed genes. **(A,B)** Distribution of m6A peaks per mRNA **(A)** or gene **(B)**. **(C)** The distributions of m6A peaks in all chromosomes after mapping to rat chromosomes. **(D)** Top 20 enrichments revealed in GO enrichment analyses of m6A modification in cardiomyocyte regeneration. **(E)** Pathway-enrichment analyses of m6A modification in P0 and P7 heart tissues. **(F–I)** The top 15 GO **(F,G)** and KEGG pathways **(H,I)** of un- or downregulated genes in P0 and P7 rat hearts after RNA-seq. GO, Gene ontology.

Supplementary Figure 2 GO and KEGG pathway analyses of significant m6A peaks in both m6A modification and RNA expression genes. **(A,D)** Top 15 GO **(A,B)** and KEGG pathways **(C,D)** for un- or downregulation in both m6A modification and RNA expression genes after conjoint analyses of MeRIP-seq and RNA-seq data.

Supplementary Figure 3 Data visualization analysis of eight hub genes. The m6A level and the expression of eight hub genes were conducted by Integrative Genomics Viewer and showed in Day 7 and Day 0.

Supplementary Figure 4 Protein expression levels of METTL3. **(A–B)** Protein expression levels of METTL3 in P0 NRCMs transfected with si-NC or si-METTL3 **(A)** and the corresponding densitometric analysis **(B)**. **(C–D)** Protein expression levels of METTL3 in P0 NRCMs transfected with adenovirus-GFP or adenovirus-METTL3 **(C)** and the corresponding densitometric analysis **(D)**.

Supplementary Table 1 List of utilized primers for qRT-PCR.

Supplementary Table 2 Most relevant networks of hyper- or hypo-methylated peaks.

Supplementary Table 3 Most relevant networks of m6A-modified genes.

Supplementary Table 4 The change of m6A and expression about miRNAs' targets in our data.
