# Correction: Comprehensive analysis of the transcriptome-wide m6A methylome of heart via MeRIP after birth: day 0 vs. day 7

**DOI:** 10.3389/fcvm.2025.1662054

**Published:** 2025-08-29

**Authors:** Chuanxi Yang, Kun Zhao, Jing Zhang, Xiaoguang Wu, Wei Sun, Xiangqing Kong, Jing Shi

**Affiliations:** ^1^ Department of Cardiology, Medical School of Southeast University, Nanjing, China; ^2^ Department of Cardiology, The First Affiliated Hospital of Nanjing Medical University, Nanjing, China

**Keywords:** m6A, epitranscriptome, heart regeneration, METTL3, cardiomyocyte

There was an error in Figure 1C and Figure 8A as published. Due to our mistake in combining images, GAPDH band in Figures 1C, and images for NRCMs staining in Figure 8A were misused. The corrected Figure 1C and Figure 8A appear below.

**Figure 1:**
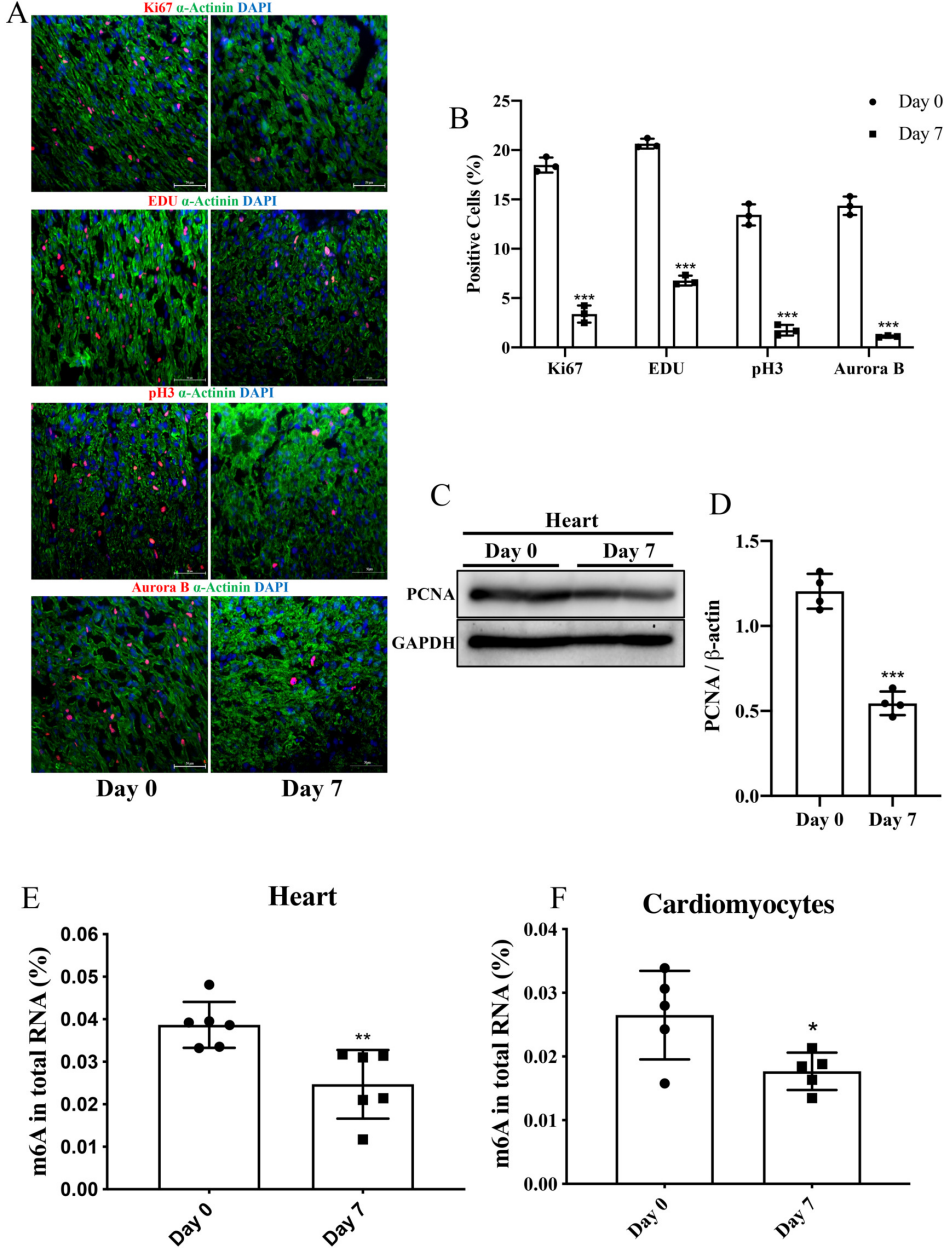
The level of m6A in P7 rat heart was decreased compared to P0. **(A,B)** Representative immunofluorescence images of paraffin-embedded heart sections labeled with α-Actinin, Ki67, pH3, Aurora B, and EDU at 200 × magnification (α-Actinin, green; Ki67, pH3, Aurora B, and EDU, red; DAPI, blue. Scale bars, 200 µm). **(C,D)** Protein expression levels of PCNA as determined by Western blotting **(C)** in heart tissue from P0 and P7 rats and the corresponding densitometric analyses **(D)** GAPDH was detected as the loading control. **(E,F)** Quantification of m6A in total RNA in heart tissue **(E)** and NRCMs **(F)** from P0 and P7 rats. N 3 per group. The results are expressed as means ± SEMs (NS indicates not significant, *P 0.05, **P 0.01, ***P 0.001, compared to the control group).

**Figure 8:**
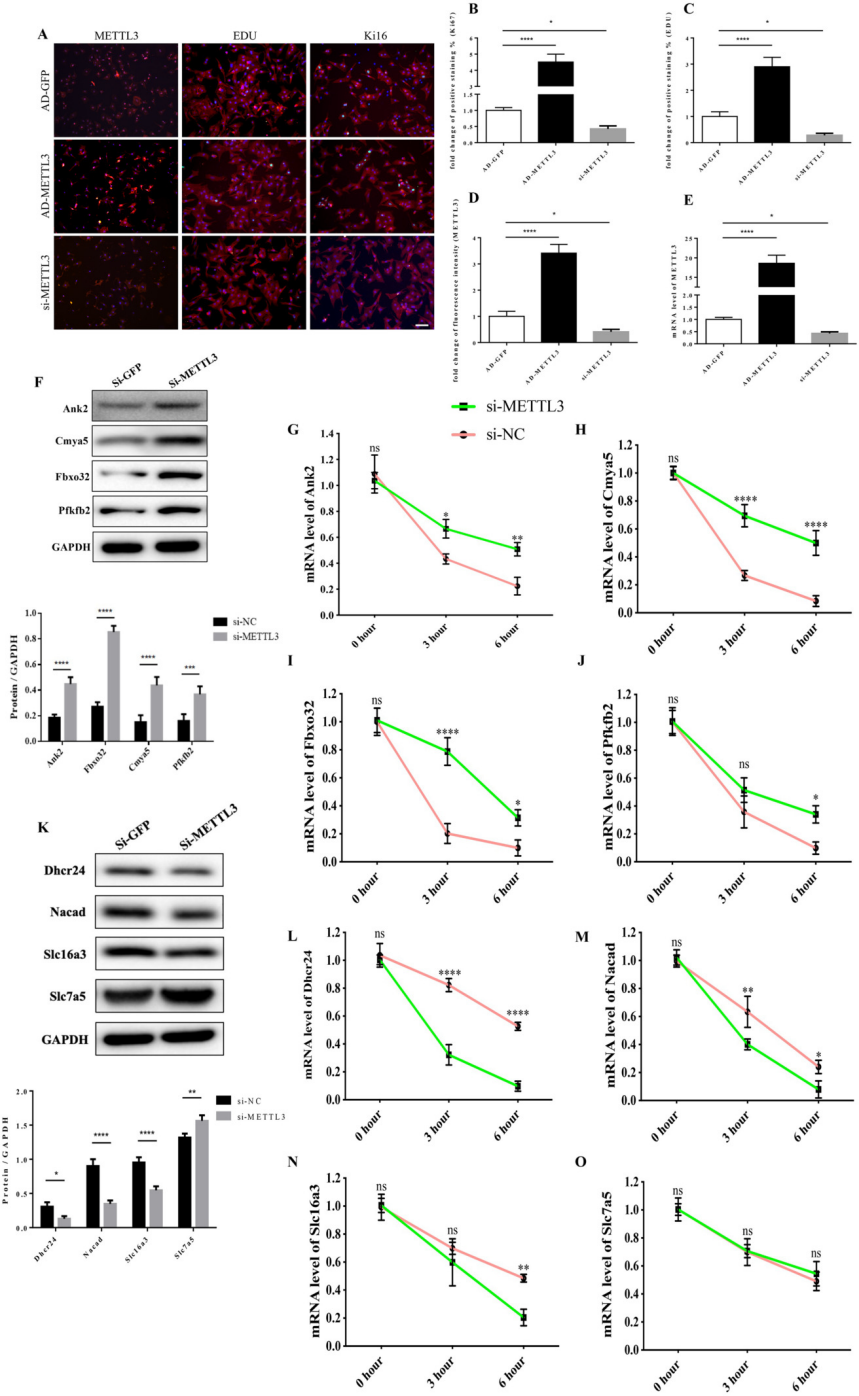
The functional link of enhanced METTL3 expression to transcript stability of target genes. **(A–D)** Representative immunofluorescence images of NRCMs from P0 rat hearts labeled with METTL3, EDU, and Ki67 (METTL3, or α-actinin, red; EDU, or Ki67, green; DAPI, blue. Scale bars, 50 µm) **(A)** and their corresponding quantitative analysis **(B–D)**. **(E)** mRNA expression level of METTL3 of NRCMs from P0 rat hearts transfected with AD-GFP, AD-METTL3, or si-METTL3 determined by the qPCR method. **(F)** Protein expression levels of Pfkfb2, Ank2, Cmya5, and Fbxo32 in P0 NRCMs transfected with si-NC or si-METTL3 (up) and the corresponding densitometric analysis (down). **(G–J)** mRNA expression level of Ank2 **(G)**, Cmya5 **(H)**, Fbxo32 **(I)**, and Pfkfb2 **(J)** in P0 NRCMs transfected with si-NC or si-METTL3 after treated with 20 µg/ml Actinomycin D for 0, 3 or 6 h. **(K)** Protein expression levels of Dhcr24, Nacad, Slc16a3, and Slc7a5 in P0 NRCMs transfected with si-NC or si-METTL3 (up) and the corresponding densitometric analysis (down). (L–O) mRNA expression level of Dhcr24 **(L)**, Nacad **(M)**, Slc16a3 **(N)**, and Slc7a5 **(O)** in P0 NRCMs transfected with si-NC or si-METTL3 after treated with 20 µg/ml Actinomycin D for 0, 3 or 6 h. GAPDH was detected as the loading control. *P 0.05, **P 0.01, ****P 0.001, *****P 0.0001, compared to the si-NC group.

File Supplementary Image 1 was erroneously published with the original version of this paper. The file has now been replaced.

The original article has been updated.

