# Supplementary figures and images for: Comprehensive Analysis of the Transcriptome-Wide m6A Methylome of Heart via MeRIP After Birth: Day 0 vs. Day 7
Source: Front Cardiovasc Med. 2021 Mar 22;8:633631. doi: 10.3389/fcvm.2021.633631 (PMC8019948; doi:10.3389/fcvm.2021.633631)

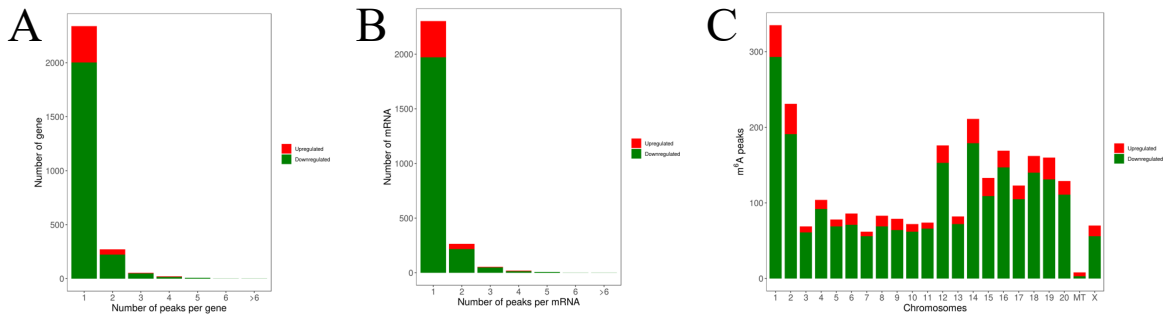

**D**

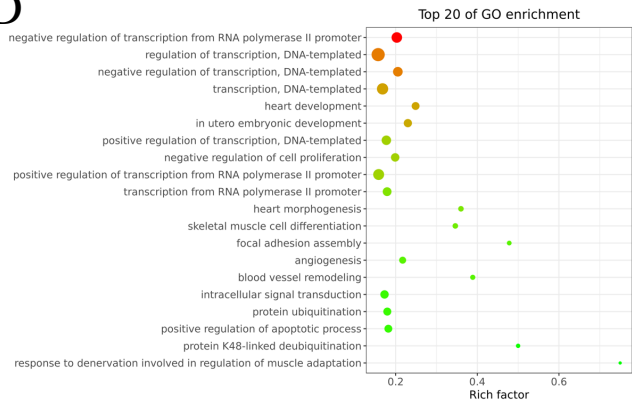

**E**

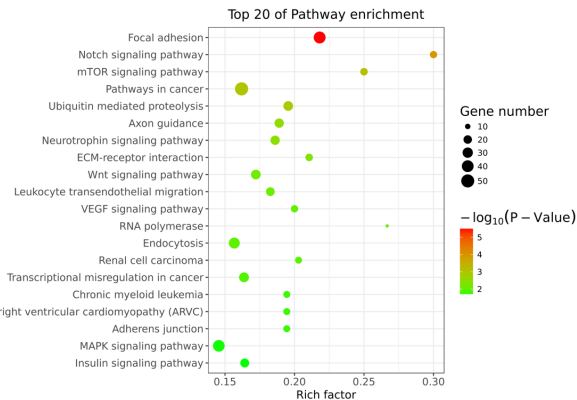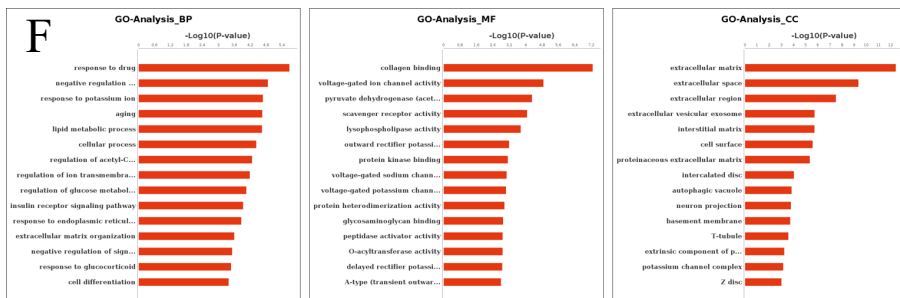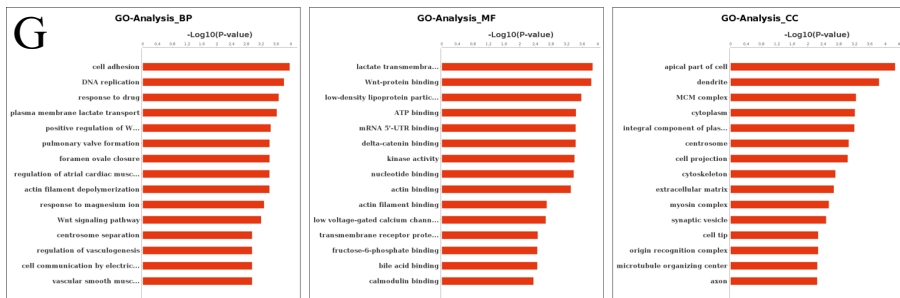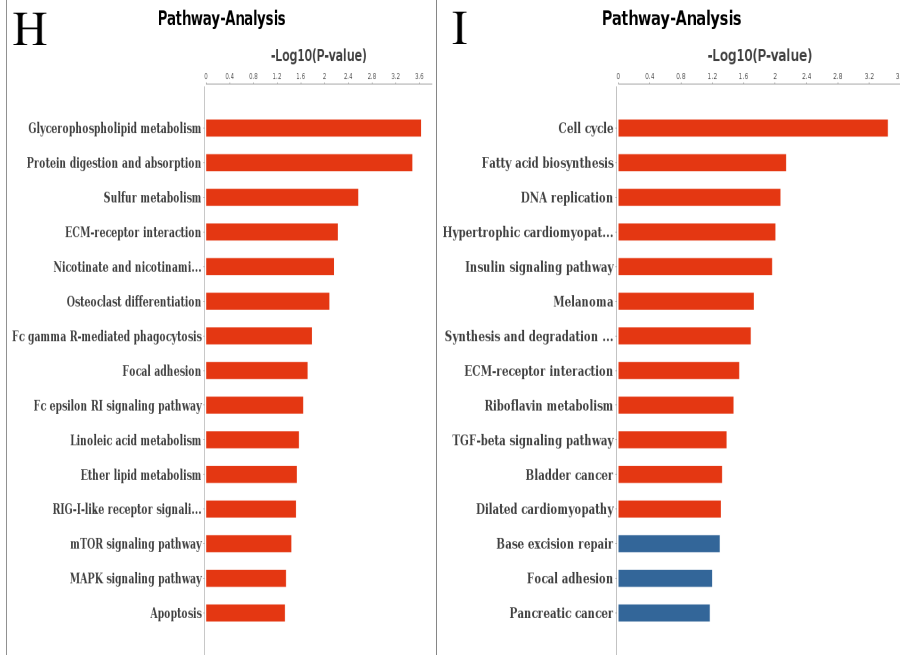

Supplement: Supplementary Figure 1 — Distribution and signaling pathways of m6A peaks and differentially expressed genes. (A,B) Distribution of m6A peaks per mRNA (A) or gene (B). (C) The distributions of m6A peaks in all chromosomes after mapping to rat chromosomes. (D) Top 20 enrichments revealed in GO enrichment analyses of m6A modification in cardiomyocyte regeneration. (E) Pathway-enrichment analyses of m6A modification in P0 and P7 heart tissues. (F–I) The top 15 GO (F,G) and KEGG pathways (H,I) of un- or downregulated genes in P0 and P7 rat hearts after RNA-seq. GO, Gene ontology. [file Image_1.PDF]

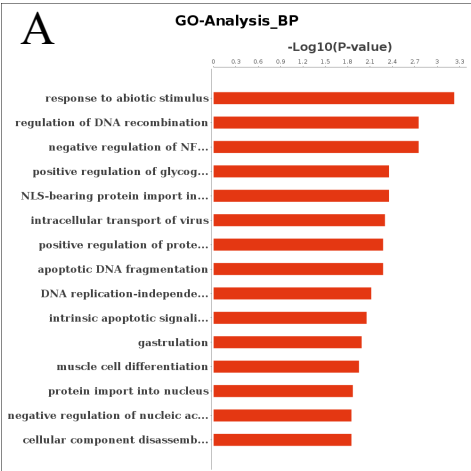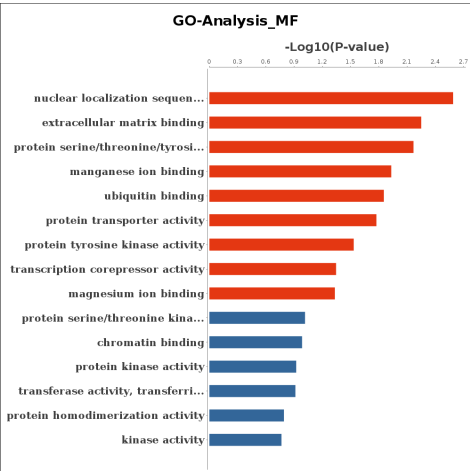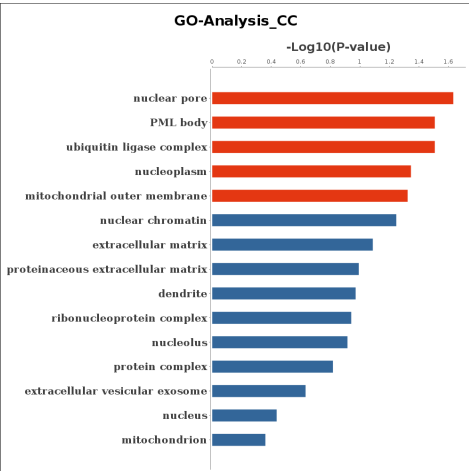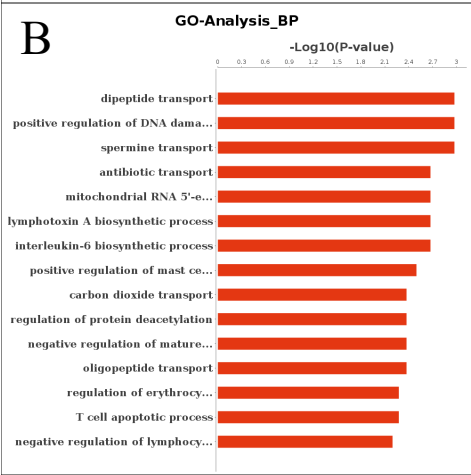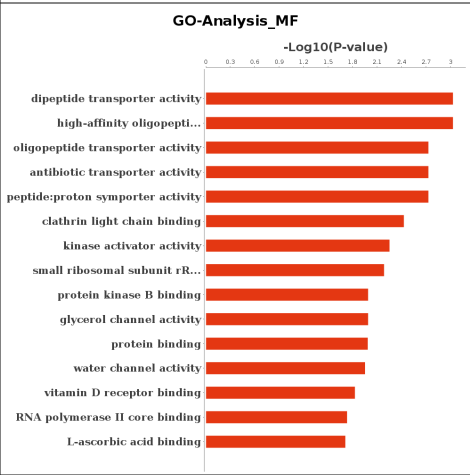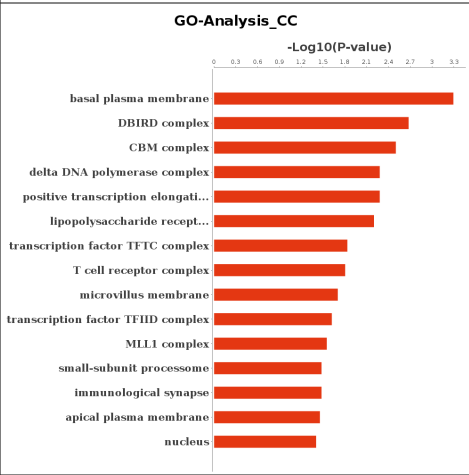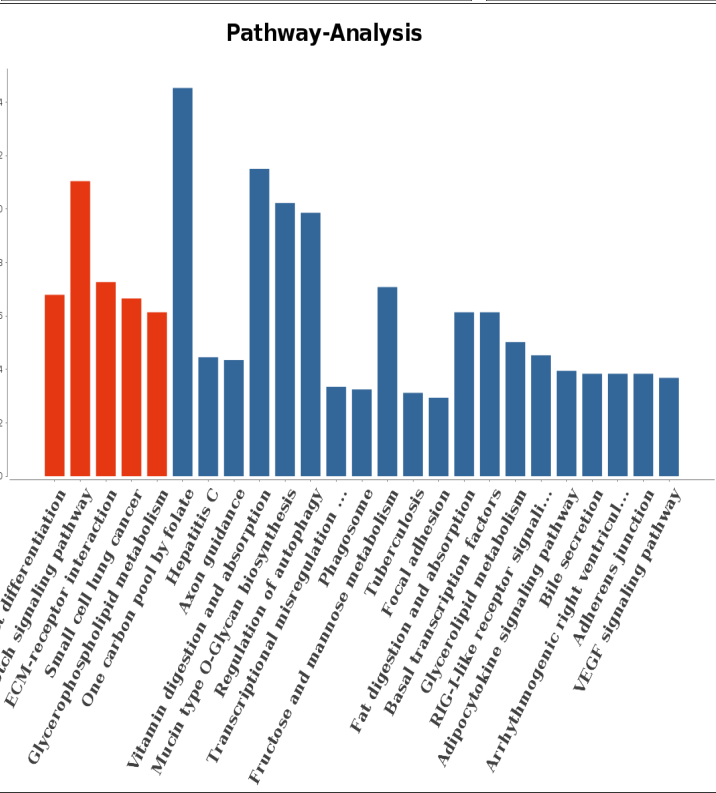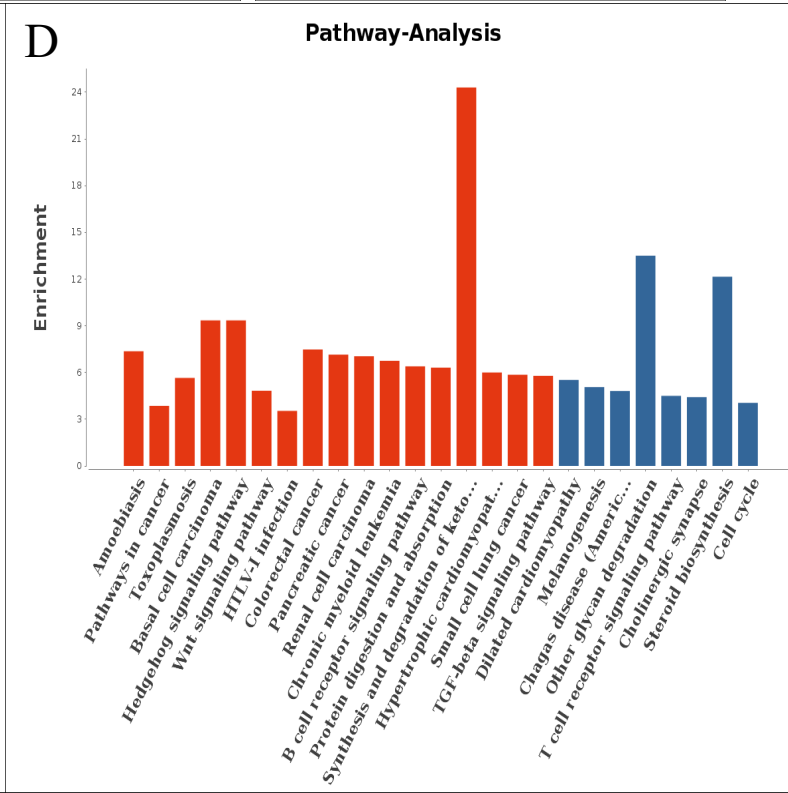

Supplement: Supplementary Figure 2 — GO and KEGG pathway analyses of significant m6A peaks in both m6A modification and RNA expression genes. (A,D) Top 15 GO (A,B) and KEGG pathways (C,D) for un- or downregulation in both m6A modification and RNA expression genes after conjoint analyses of MeRIP-seq and RNA-seq data. [file Image_2.PDF]

## Ank2

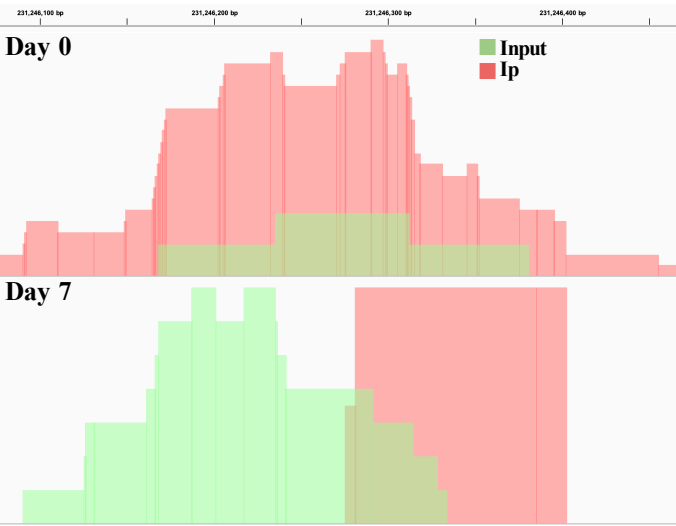

## Slc7a5

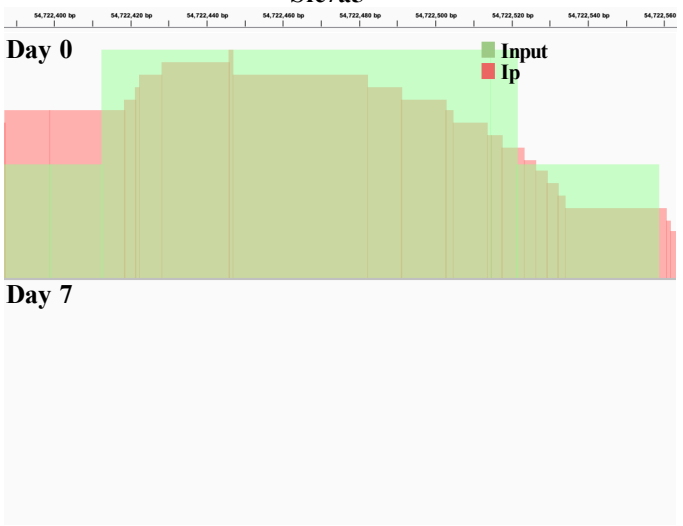

## Fbxo32

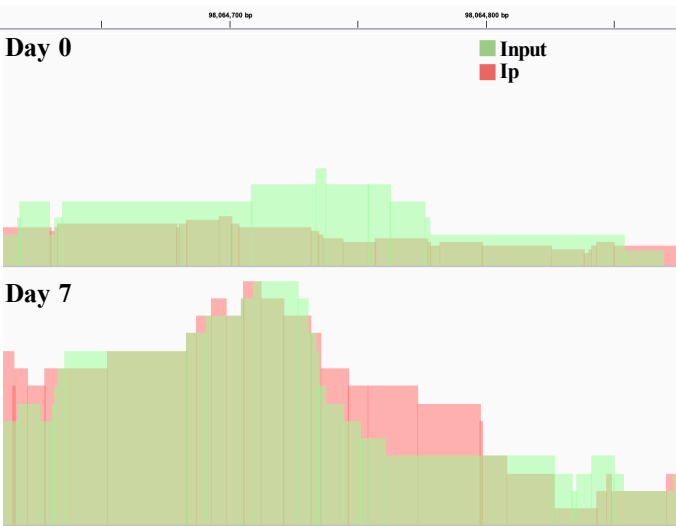

## Pfkfb2

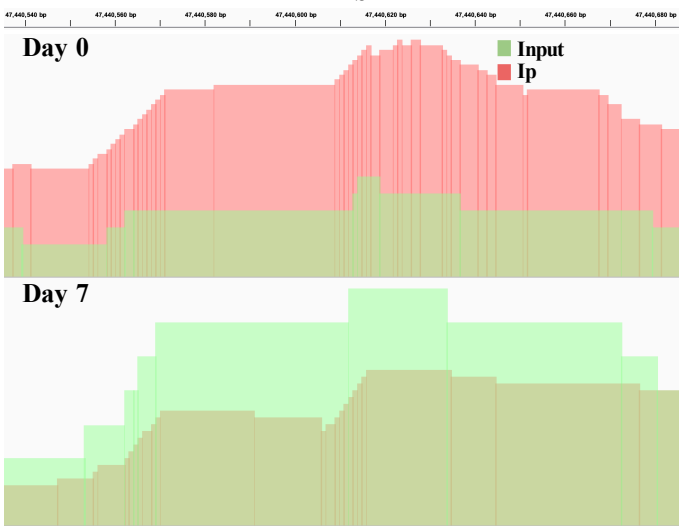

## Nacad

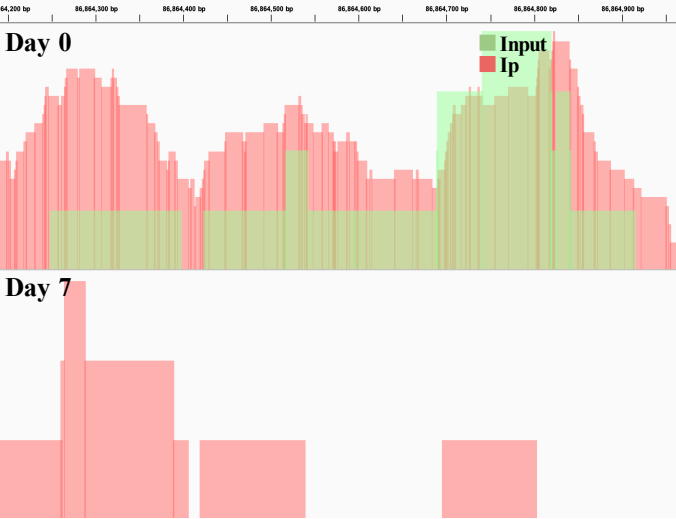

## Slc16a3

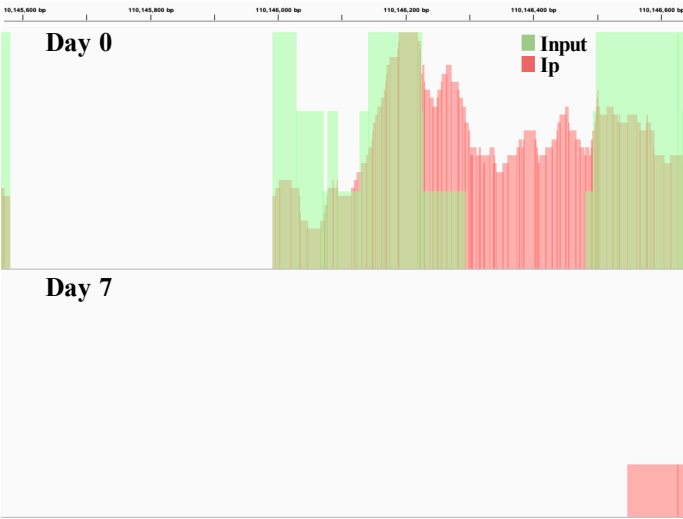

## Dhcr24

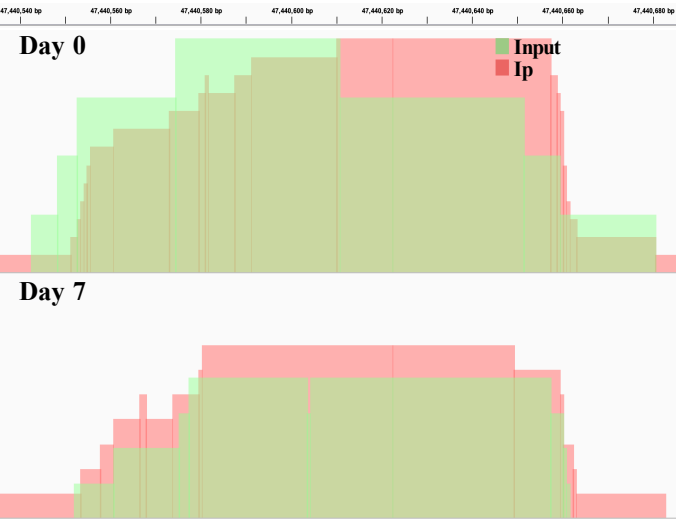

## Cmya5

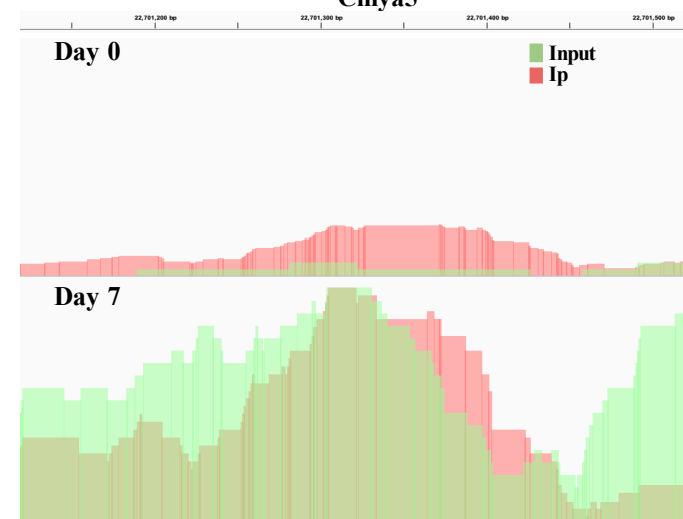

Supplement: Supplementary Figure 3 — Data visualization analysis of eight hub genes. The m6A level and the expression of eight hub genes were conducted by Integrative Genomics Viewer and showed in Day 7 and Day 0. [file Image_3.PDF]

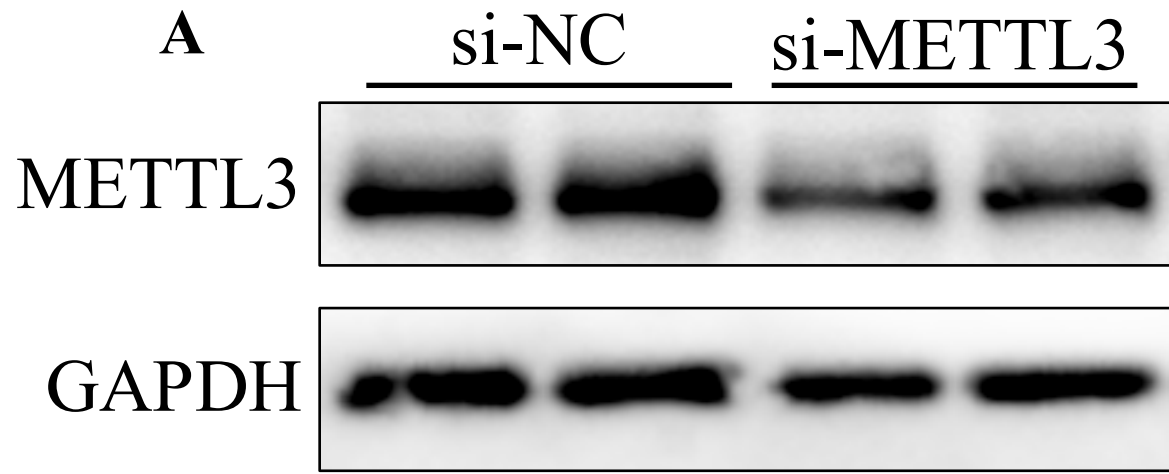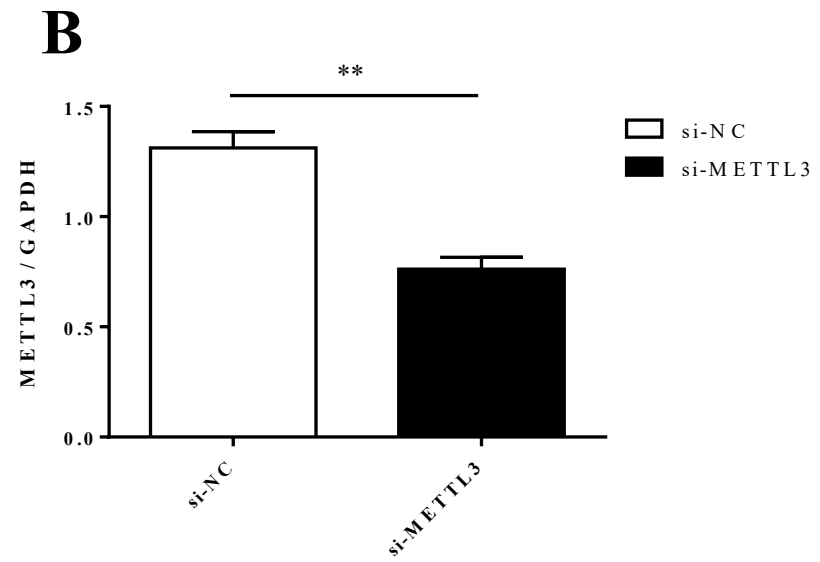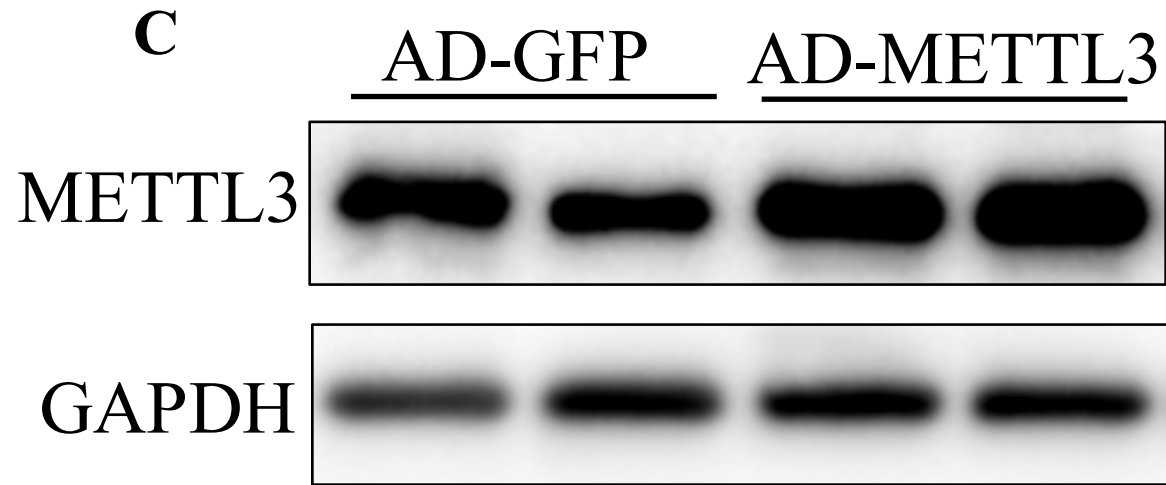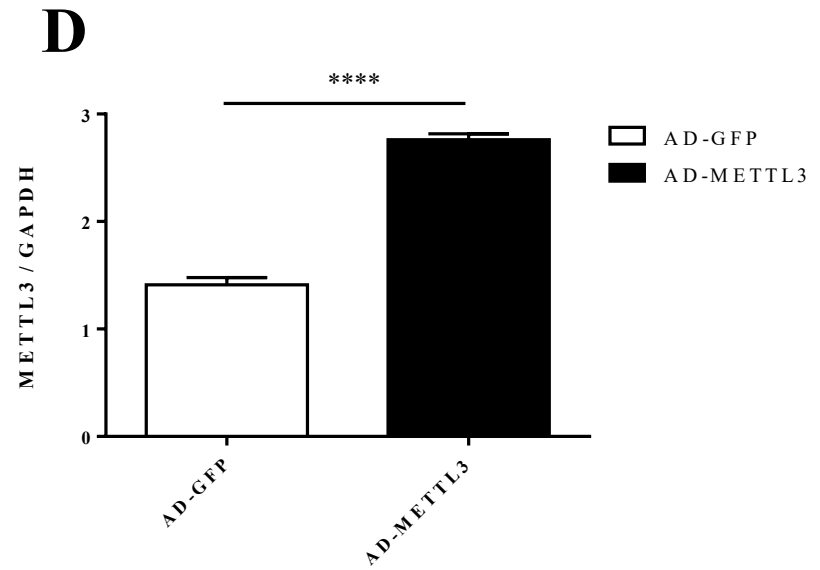

Supplement: Supplementary Figure 4 — Protein expression levels of METTL3. (A–B) Protein expression levels of METTL3 in P0 NRCMs transfected with si-NC or si-METTL3 (A) and the corresponding densitometric analysis (B). (C–D) Protein expression levels of METTL3 in P0 NRCMs transfected with adenovirus-GFP or adenovirus-METTL3 (C) and the corresponding densitometric analysis (D). [file Image_4.PDF]
